# Evidence-Based Annotation of Gene Function in *Shewanella oneidensis* MR-1 Using Genome-Wide Fitness Profiling across 121 Conditions

**DOI:** 10.1371/journal.pgen.1002385

**Published:** 2011-11-17

**Authors:** Adam Deutschbauer, Morgan N. Price, Kelly M. Wetmore, Wenjun Shao, Jason K. Baumohl, Zhuchen Xu, Michelle Nguyen, Raquel Tamse, Ronald W. Davis, Adam P. Arkin

**Affiliations:** 1 Physical Bioscience Division, Lawrence Berkeley National Laboratory, Berkeley, California, United States of America; 2 Earth Sciences Division, Lawrence Berkeley National Laboratory, Berkeley, California, United States of America; 3 Department of Bioengineering, University of California Berkeley, Berkeley, California, United States of America; 4 Stanford Genome Technology Center, Department of Biochemistry, Stanford University, Stanford, California, United States of America; Progentech, United States of America

## Abstract

Most genes in bacteria are experimentally uncharacterized and cannot be annotated with a specific function. Given the great diversity of bacteria and the ease of genome sequencing, high-throughput approaches to identify gene function experimentally are needed. Here, we use pools of tagged transposon mutants in the metal-reducing bacterium *Shewanella oneidensis* MR-1 to probe the mutant fitness of 3,355 genes in 121 diverse conditions including different growth substrates, alternative electron acceptors, stresses, and motility. We find that 2,350 genes have a pattern of fitness that is significantly different from random and 1,230 of these genes (37% of our total assayed genes) have enough signal to show strong biological correlations. We find that genes in all functional categories have phenotypes, including hundreds of hypotheticals, and that potentially redundant genes (over 50% amino acid identity to another gene in the genome) are also likely to have distinct phenotypes. Using fitness patterns, we were able to propose specific molecular functions for 40 genes or operons that lacked specific annotations or had incomplete annotations. In one example, we demonstrate that the previously hypothetical gene SO_3749 encodes a functional acetylornithine deacetylase, thus filling a missing step in *S. oneidensis* metabolism. Additionally, we demonstrate that the orphan histidine kinase SO_2742 and orphan response regulator SO_2648 form a signal transduction pathway that activates expression of acetyl-CoA synthase and is required for *S. oneidensis* to grow on acetate as a carbon source. Lastly, we demonstrate that gene expression and mutant fitness are poorly correlated and that mutant fitness generates more confident predictions of gene function than does gene expression. The approach described here can be applied generally to create large-scale gene-phenotype maps for evidence-based annotation of gene function in prokaryotes.

## Author Summary

Many computationally predicted gene annotations in bacteria are incomplete or wrong. Consequently, experimental methods to systematically determine gene function in bacteria are required. Here, we describe a genetic approach to meet this challenge. We constructed a large transposon mutant library in the metal-reducing bacterium *Shewanella oneidensis* MR-1 and profiled the fitness of this collection in more than 100 diverse experimental conditions. In addition to identifying a phenotype for more than 2,000 genes, we demonstrate that mutant fitness profiles can be used to assign “evidence-based” gene annotations for enzymes, signaling proteins, transporters, and transcription factors, a subset of which we verify experimentally.

## Introduction

Advances in sequencing technology have ushered in a new era of bacterial genomics. At low cost, a single individual can sequence the complete genome of a bacterial isolate in less than a week. While this explosion in genome sequencing and gene discovery is breathtaking, it also serves as a reminder that for most bacteria, we do not know the function for most of the genes in the genome [1]. Even in the model bacterium *Escherichia coli*, which has been extensively studied for decades, there are hundreds of genes that are poorly annotated or entirely hypothetical [2]. Therefore, it is critical that methods for systematically elucidating gene function in microbial genomes are developed [3].

The current paradigm is that newly sequenced bacterial genomes go through a computational annotation pipeline that predicts gene structure and putative function. The latter is predicted from sequence homology to known gene families, protein domains, and characterized enzymes. However, given that most experimentally characterized genes derive from a small number of bacteria representing a tiny fraction of prokaryotic diversity, there are a large number of gene families that have never been experimentally characterized and hence computational annotations are useless beyond “conserved domain” or “conserved hypothetical.” Furthermore, due to weaker sequence conservation, computational annotations of gene function in microbial species get progressively less reliable the further one moves away from well-studied model bacteria such as *E. coli* and *Bacillus subtilis* [4]. Lastly, there are classes of genes (for instance, transcription factors [5] and transport proteins [6]) for which homology-based annotations are either vague or unreliable. Taken together, computational predictions alone, while a necessary first step towards genome annotation, are not sufficient to meet the growing challenge of assigning function to the millions of genes identified by DNA sequencing.

One attractive approach to characterize genes on a global scale is via the analysis of large-scale mutant collections. Mutants provide insight into gene function by providing a direct link between genotype and a cellular phenotype. By correlating genes with their phenotypes, a specific gene function can often be inferred [7], [8], [9], [10]. In the post-genome era, a number of microorganisms have been subjected to large-scale mutagenesis and phenotyping efforts. In bacteria, genome-wide mutant collections have been constructed for several bacteria, using either targeted methods [11], [12], [13] or random transposon mutagenesis [14], [15], [16].

One attractive approach to characterize genes on a global scale is via the analysis of large-scale mutant collections. Mutants provide insight into gene function by providing a direct link between genotype and a cellular phenotype. By correlating genes with their phenotypes, a specific gene function can often be inferred [7], [8], [9], [10]. In the post-genome era, a number of microorganisms have been subjected to large-scale mutagenesis and phenotyping efforts. In bacteria, genome-wide mutant collections have been constructed for several bacteria, using either targeted methods [11], [12], [13] or random transposon mutagenesis [14], [15], [16].

Regardless of how mutant strains are generated, a key challenge is the quantitative analysis of the mutant collections across the diverse range of conditions necessary to identify phenotypes for the majority of genes in the genome [17]. The phenotypes of mutant collections can be assayed in high-throughput either as individual strains or in pooled, competitive fitness assays. In a recent example of the former approach, the individual mutant strains of the *E. coli* KEIO deletion collection were assayed in hundreds of growth conditions using an agar-based colony size assay [10]. At least one phenotype was identified for ∼50% of *E. coli* genes using this individual strain assay. Conversely, the use of pooled assays to measure mutant phenotypes is best exemplified in *Saccharomyces cerevisiae*. Each yeast deletion strain contains a unique DNA tag (or barcode) sequence, that enables the pooling and competitive fitness profiling of thousands of strains in parallel [8]. Similar to individual strain assays, competitive pool assays provide a relative measure of strain fitness. Nevertheless, the use of competitive fitness assays with DNA tags has two primary advantages. The first is that genome-wide mutant collections are pooled in a single tube, thereby simplifying experimental setup, increasing throughput, and reducing issues related to strain contamination. More importantly, the tag-based pooled fitness assay provides a highly quantitative measure of strain fitness regardless of whether a microarray [18], [19] or sequencing [20] is used to measure tag abundance.

*Shewanella oneidensis* MR-1 is a Gram-negative γ-proteobacterium isolated from freshwater lake sediment [21]. Like most other members of the *Shewanella* genus, *S. oneidensis* MR-1 (hereafter abbreviated MR-1) can use a wide variety of terminal electron acceptors, including both soluble and solid metals. As such, MR-1 has received attention for its potential roles in the bioremediation of heavy metals and energy generation via fuel cells [22]. The computationally annotated MR-1 genome contains 4,318 protein-coding genes on its main chromosome and an additional 149 protein-coding genes on a single megaplasmid [23], [24]. Based on orthology relationships (bidirectional best BLAST hits), MR-1 shares 1,639 genes (37%) with the γ-proteobacterium *E. coli*. A total of 1,655 genes (37%) in the MR-1 genome are annotated as hypothetical, with 83% (1,371) of these genes not having orthologs in *E. coli*.

Here we describe the functional characterization of the MR-1 genome via the generation and phenotypic analysis of a large transposon mutant collection. Using a DNA tag-based pooled fitness assay, we assayed mutant fitness for 3,355 nonessential genes in 121 diverse metabolic, redox, stress, survival, and motility conditions. In addition to identifying phenotypes for over 2,000 genes, we demonstrate that mutant fitness profiles can be used to infer specific functions for genes and operons, a subset of which we confirm experimentally. Furthermore, we demonstrate that the correlation between gene expression and mutant fitness is poor in bacteria, thus underscoring the need to complement transcriptomics with mutant phenotyping. Our strain collection and fitness dataset are valuable resources for studying microbial metal reduction and for microbiology in general, given that many previously uncharacterized MR-1 genes have orthologs in diverse bacteria.

## Results

### A *S. oneidensis* MR-1 fitness compendium

To provide a deep functional characterization of the MR-1 genome by analysis of mutant phenotypes, we expanded our previously characterized transposon library [19] by mapping an additional 17,301 mutants. The total MR-1 transposon collection consists of 24,688 archived strains and represents mutants in 3,447 unique genes (Table S1). Genes without a transposon insertion are potentially essential for viability. Starting from this premise, we classified 336 MR-1 genes as ExpectedEssential because their orthologs are essential in *E. coli* [25], [26] and 67 NewEssential genes that were not expected from studies of *E. coli*, including 12 that are not orthologs of essential genes in any of 14 other bacteria (DEG version 5.4) [27]. The 67 NewEssential genes include those of the ATP synthase complex, gluconeogenesis, biotin synthesis, and phosphate transport (see Text S1 for full analysis; Table S2 for full essential gene list).

**Figure 1:**
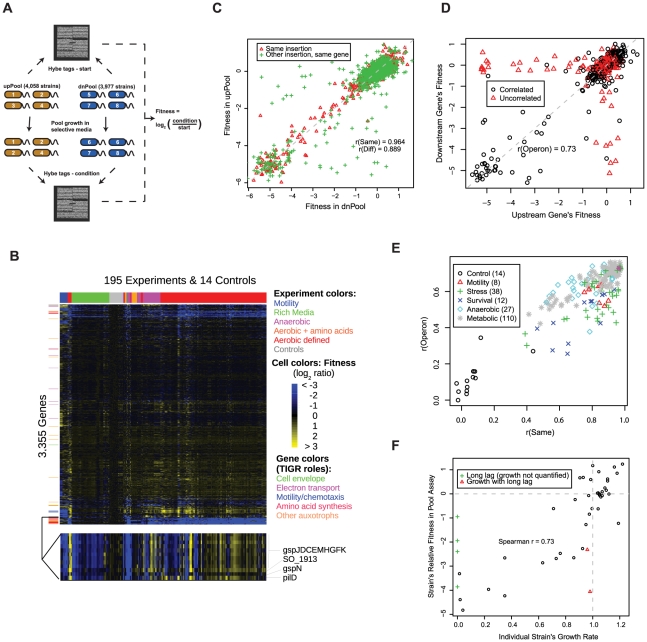
A *S. oneidensis* MR-1 mutant fitness compendium. (A) Parallel analysis of MR-1 mutant pools using TagModules. Only the uptags are used to interrogate strain abundance in the upPool, while the downtags are used exclusively to measure strain abundance in the dnPool. The Affymetrix TAG4 microarray illustrated here contains the complement sequences to both the uptags and downtags, therefore the abundance of all strains across both pools is assayed in a single hybridization. In the simple example diagrammed here, strain 3 in the upPool and strain 5 in the dnPool have fitness defects. We hybridize the tags both before (start) and after growth in selective media (condition). We calculate the fitness of a strain as the normalized log_2_ ratio of tag intensity of the condition relative to start. (B) Heatmap of the entire fitness dataset. Both genes and experiments were ordered by hierarchical clustering with Euclidean distance as the metric. A subset of the fitness heatmap for mutants in the general secretory pathway is expanded (bottom). (C) Fitness values for dnPool strains (x-axis) and upPool strains (y-axis) on DL-lactate defined media. “Same insertion” indicates identical mutant strains that are represented in both pools; “Other insertion, same gene” indicates independent transposon insertions in the same gene. The dashed line shows x = y. (D) Comparison of gene fitness values for pairs of genes predicted to be in the same operon [50]. The data plotted reflects a single fitness experiment in DL-lactate minimal media, but the color-coding is derived from the entire fitness compendium: points in red are uncorrelated (r<0.3) across 195 fitness experiments. (E) Quality metrics for each of the 195 pool fitness experiments. r(Same) is the fitness correlation of identical mutant strains contained in both the upPool and dnPool (see red triangles in panel C). r(Operon) is the fitness correlation of adjacent genes predicted to the in the same operon (see panel D). (F) Comparison of DL-lactate minimal media pool fitness values (y-axis) and individual strain growth rates (x-axis) for 48 transposon mutants. Individual strain growth rates represent the average of at least three independent experiments. The vertical dotted line represents the growth rate of wild-type MR-1. The horizontal dotted line represents a pool fitness value for a neutral insertion. Some strains had long lag phases and a growth rate could not be calculated (green plus symbols).

To enable large-scale phenotypic analysis of the MR-1 mutant collection, we engineered our transposons to contain TagModules. A TagModule contains two unique 20 bp DNA tags (or barcodes), termed the uptag and downtag, each flanked by common PCR priming sites. In a system identical to that used for the yeast deletion collection, thousands of mutant strains, each carrying a unique TagModule, can be pooled together and competitively grown in a condition of interest [9], [19]. We calculated the fitness of each strain as the log_2_ ratio of the signal for the tag after growth in that condition relative to the start of the experiment (Figure 1A). Negative log_2_ ratios indicate that the given strain has a fitness defect relative to the median strain; positive log_2_ ratios indicate a fitness advantage. We constructed two pools, the upPool and dnPool, for the phenotypic analysis of MR-1 mutants (Figure 1A). These pools contain transposon mutants in 3,355 MR-1 genes (Figure S1).

To assess the potential of mutant fitness to annotate gene function, we assayed the fitness of the pooled strains in 195 experiments and 121 different conditions (see Table S3). These conditions include aerobic experiments in defined minimal media with one of 26 different sole sources of carbon, 20 for nitrogen, 8 for sulfur, and 5 for phosphorous. Given the unique activity of *Shewanella* with respect to anaerobic respiration, we also profiled the pool fitness under anaerobic conditions with different electron acceptors including iron (III) citrate, manganese (IV) oxide, fumarate, dimethyl sulfoxide (DMSO), and nitrate. Additionally, we profiled a number of stresses including metals, salts, temperature, pH, stationary phase, and heat shock. Lastly, we assayed motility using a soft agar assay. A heatmap of our entire fitness compendium is presented in Figure 1B.

### Quality assessment of fitness compendium

We used a number of tests to validate the technical and biological consistency of our mutant fitness dataset including the correlation of related strains or genes, the fitness of expected auxotrophs in rich versus minimal media, the relationship between fitness values and growth rates when grown individually, and complementation of mutant phenotypes. First, we compared the fitness for (1) identical mutant strains contained in both pools but assayed with different tags (uptag for upPool and downtag for dnPool) and (2) different transposon mutants for the same gene (Figure 1C). For the identical mutant strains, the fitness values are highly correlated when assayed in the upPool versus the dnPool (r = 0.96; Pearson correlation), which confirms that a single uptag or downtag of a TagModule is sufficient for measuring fitness. The fitness correlation is slightly lower (r = 0.89; Pearson correlation) for different mutants in the same gene (Figure 1C). Next, we examined the cofitness (correlation of fitness) of operons, cotranscribed groups of genes that are often functionally related [28]. We defined the fitness of a gene as the average of relevant strain fitness values (see Materials and Methods). As illustrated in Figure 1D, our data confirms the expectation that the fitness of genes within an operon are positively correlated in a single condition (r = 0.73; Pearson correlation). For each of our 195 pool fitness experiments, we used both the operon pair fitness correlation (as in Figure 1D) and the fitness correlation of identical strains in the upPool and dnPool (red triangles in Figure 1C) as computational metrics of experimental quality (Figure 1E).

To validate the biological consistency of our pool fitness assay, we compared gene fitness on DL-lactate minimal media to LB rich media to confirm MR-1 auxotrophs predicted by TIGR roles [29] and flux balance analysis [30]. As illustrated in Figure S2, most predicted auxotroph genes have severe fitness defects in minimal media and normal growth in rich media, which demonstrates that our pooled fitness results are biologically meaningful. For example, of 60 auxotrophs predicted by both TIGR and the flux balance analysis, 88% have fitness of −2 or less in minimal media, versus just 3% in LB rich media. Lastly, we compared the pooled fitness data of MR-1 grown in minimal media to single strain growth data of *E. coli* deletion strains grown in minimal media [11] to examine the phenotypic consistency of orthologous auxotrophic genes (Figure S3). The majority of experimentally verified MR-1 auxotrophs are also *E. coli* auxotrophs. However, there are a number of disagreements due primarily to redundancy (presence of isozymes or alternative pathways) in MR-1 but not *E. coli* (*glnA*, *metL*, *purA*) or vice versa (*panE*, *asnB*, *trxB*, *gltBD*, *argI*). MR-1 auxotrophs without *E. coli* orthologs include diverged *purC*, *aroQ*, and *folK* genes and SO_3749, a new arginine synthesis gene (see below).

It is important to note that fitness as used in this study reflects the abundance of tags in a pooled assay and not an absolute growth rate for each individual strain. Hence, our pooled fitness assay does not distinguish whether less fit strains are due to slower doubling times or longer lag phases. To test this, we measured growth for 48 individual mutant strains in DL-lactate minimal media. As illustrated in Figure 1F, we observe a positive correlation (r = 0.73; Spearman correlation) between pool relative fitness values and single strain growth rates. This highlights the quantitative nature of our competitive growth assay, as previously observed in yeast [18]. Additionally, most mutants with fitness defects in the pool assay are due to slower doubling times rather than extended lag phases (Figure 1F).

**Figure 2:**
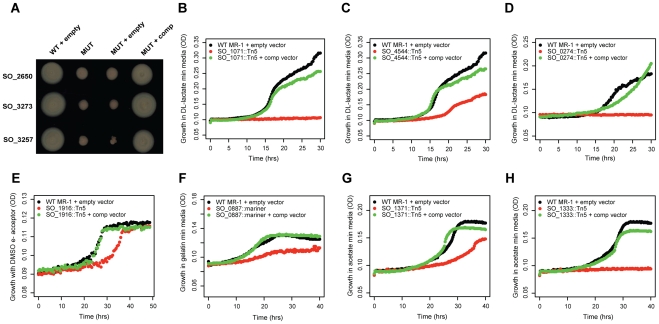
Validation of mutant phenotypes by genetic complementation. (A) Three conserved hypothetical genes are required for motility in an LB soft agar assay. MUT is a transposon mutant; MUT + empty is a transposon mutant carrying an empty plasmid, MUT + comp is a transposon mutant with a plasmid carrying an intact copy of the mutated gene. SO_2650 has no known domains. SO_3273 (protein of unknown function DUF115) contains a tetratricopeptide-like helical domain (IPR011990), a common structural motif. SO_3257 is discussed in the main text. (B) SO_1071 is a predicted membrane protein from an uncharacterized protein family (UPF0016). It is required for growth in minimal media with DL-lactate as a carbon source. (C) Same as (B) for SO_4544, a hypothetical protein with no known domains. (D) Same as (B) for SO_0274 (*ppc*) encoding phosphoenolpyruvate carboxylase. (E) SO_1916, a transcriptional regulator, is required for maximal anaerobic growth with DL-lactate as a carbon source and DMSO as an electron acceptor (also see Figure S4). (F) SO_0887, annotated as agmatine deiminase, is required for maximal growth on minimal media with gelatin as a carbon source. (G) SO_1371 is a conserved hypothetical gene, contains an RDD domain (one arginine and two aspartates), and is a predicted membrane protein. It is required for maximal growth on minimal media with acetate as a carbon source. (H) Same as (G) for SO_1333, a conserved hypothetical gene. SO_1333 is a distant homolog of the sulfoacetate transporter TauE [79].

To verify that our mutant phenotypes are caused by a single transposon insertion, we complemented the fitness defect of 10 genes, 7 of which are annotated as hypothetical (Figure 2). In each instance, we complemented the mutant phenotype by introducing an intact copy of the gene on a plasmid. These results demonstrate that poorly characterized genes have phenotypes in a diverse range of conditions and that a single transposon insertion was responsible for the fitness defect. Of note, SO_3257 is adjacent to a large flagellar/chemotaxis gene cluster in MR-1 and is a distant homolog of *Vibrio cholerae flgO* [31] (24% identity but at a conserved location) (Figure 2A). SO_0274 (*ppc*) encodes phosphoenolpyruvate carboxylase and is absolutely required for growth on minimal media with DL-lactate as a carbon source (Figure 2D). Interestingly, a flux balance model predicts that the loss of *ppc* will lead to only a 5% reduction in growth rate [30]. This discrepancy between a model prediction and an experimental result highlights the utility of fitness profiling to discover unexpected roles for characterized enzymes. Overall, these complementation results and the fitness agreement of independent mutants in the same gene (Figure 1B) suggest that secondary mutations are not a significant factor in our experiments, allowing us to use pooled fitness results as a proxy for single mutant phenotypes.

Lastly, we examined the potential role of polarity in our fitness dataset. Polarity, whereby a mutation in an upstream gene in an operon causes a loss of expression of downstream genes, is a concern in all transposon mutant studies. To address this issue, we looked for instances in which only the upstream or downstream gene of an operon pair has a strong fitness defect (fitness <−2 vs. >−1). If there are strong polar effects, then we should rarely see cases where only the downstream gene in the pair is sick, because mutating the upstream gene should usually impair the downstream gene, but not vice versa. Across our entire fitness compendium, upstream-only sick occurs only about 50% more often than downstream-only sick (3,333 vs. 2,233 cases, P<1e-15, binomial test) suggesting that most within operon fitness correlations reflect similar biological roles and are not simply an artifact of polarity. To further support the notion that polarity is not a dominant factor in our results, we successfully complemented the fitness defects of 4 upstream genes in operons (SO_0887, SO_1333, SO_3257, SO_4485; Figure 2 and Table 1).

**Table 1:** New, expanded, and confirmed *S. oneidensis* MR-1 gene annotations.

Name	VIMSS	New Annotation^1^	Class	Evidence^2^
SO_0002	199199	Glutathione uptake transporter	New	Multiple mutants
SO_0444	199636	Copper/zinc efflux protein	New	Complementation
SO_0455:0456	199647-8	Alpha-ketoglutarate transporter	New	Multiple mutants
SO_0625	199813	Cytochrome c oxidase regulatory protein	New	
SO_0888	200074	N-carbamoyl-putrescine amidase	New	Complementation;Multiple mutants
SO_1033:1034	200216-7	Vitamin B12 transporter components	Confirmed	Multiple mutants
SO_1115	200295	Glycine-aspartate dipeptidase	Expanded	Multiple mutants
SO_1267	200445	Gamma-glutamyl-aminobutyrate hydrolase	Confirmed	
SO_1268	200446	Gamma-glutamyl-putrescine synthetase	Confirmed	
SO_1270:1273	200448-51	Broad range amino acid transporter	Expanded	Multiple mutants
SO_1427:1432	200602-7	DMSO or manganese oxide reductase	Expanded	Multiple mutants
SO_1521	200692	D-lactate:flavin oxidoreductase	New	
SO_1670	200835	Fumarylacetoacetate hydrolase	Confirmed	Complementation
SO_1677	200842	Acetyl-CoA/2-methyl-acetyl-CoA acetyltranferase	Expanded	Multiple mutants
SO_1679	200844	Methylbutyrl-CoA oxidoreductase	Confirmed	Multiple mutants
SO_1683	200848	Putative 2-methyl-3-hydroxybutyryl-CoA dehydrogenase	New	
SO_1854	201016	Outer membrane protein required for motility and nitrate resistance	New	Multiple mutants
SO_1913	201074	Chaperone for general secretion pathway	New	Multiple mutants
SO_1916	201077	DMSO-specific transcriptional activator of SO_1917	New	Complementation;Gene expression
SO_1971	201132	Butyryl-CoA synthase	New	Multiple mutants
SO_2357	201501	Cytochrome c oxidase maturation protein	New	Multiple mutants
SO_2395	201539	Butyryl-CoA dehydrogenase	New	Multiple mutants
SO_2593	201733	NAD amino acid dehydrogenase	Expanded	Multiple mutants
SO_2638	201776	Branched-chain amino acid dehydrogenase	Confirmed	Multiple mutants
SO_2648	201786	Response regulator and transcriptional activator of Acetyl-CoA synthase	New	Gene expression
SO_2742	201880	Histidine kinase targeting SO_2648	New	Gene expression
SO_2846	201972	Glycine transport protein	New	Multiple mutants
SO_2879	202005	N-acetylglucosamine and uracil permease	New	
SO_3102:3103	202218-9	Thiophosphate efflux pump components	New	Multiple mutants
SO_3175	202285	Cell wall component synthesis enzyme	New	
SO_3259:3260	202367-8	Flagellar modification gene cluster	New	Multiple mutants
SO_3262	202370	Polysaccharide synthesis gene	New	Multiple mutants
SO_3496	202599	Succinate-semialdehyde:NAD dehydrogenase	Confirmed	Multiple mutants
SO_3635	202731	Cell wall phosphotransferase required for survival in late stationary phase	New	Multiple mutants
SO_3749	202842	N-acetyl-L-ornithine deacetylase	New	Complementation,Multiple mutants
SO_4008	203092	Recombination regulatory protein	New	
SO_4198	203280	Formiminoglutaminase	Confirmed	Complementation
SO_4339	203417	Hypotaurine transporter	New	Multiple mutants
SO_4485	203560	Diheme cytochrome c for energy production	New	Complementation
SO_4565	203636	L-leucine transporter	New	Multiple mutants

1 See Text S2 for details.

2 Complementation indicates that a plasmid-carried, intact copy of the mutated gene rescued the relevant mutant phenotype. Multiple mutants mean that, for the indicated gene or operon, our fitness dataset contains data for two or more independent mutant strains. Gene expression indicates that microarray gene expression of the mutant strain was used to validate the prediction.

### Identifying a phenotype for more than 2,000 genes in *S. oneidensis* MR-1

Our fitness dataset provides an opportunity to explore general principles related to the phenotypic importance of single genes across a large number of diverse conditions. First, we sought to determine how many genes have at least one phenotype (positive or negative). We used the 14 control experiments (independent pool recovery experiments from the freezer or “start” in Figure 1A; grey bar in Figure 1B, black circles in Figure 1E) to transform a test statistic, which quantifies the consistency of the various measurements of a gene's fitness, to a Z score (see Materials and Methods for details). In other words, the Z scores from the control experiments follow the standard normal distribution and Z scores in the other experiments indicate their level of statistical significance. To test whether a gene's pattern of fitness is consistent with it having no phenotype, we used a chi-squared test to combine the Z scores. By combining the Z scores from all experiments, we were able to detect significant fitness patterns for genes with mild phenotypes in many conditions. At P<0.001 (chi-squared test), we find that 2,350 genes have a statistically significant fitness pattern. Therefore, 70% of the MR-1 nonessential genome (2,350 genes with a phenotype out of 3,355 assayed) has a phenotypic consequence when disrupted by a transposon.

**Figure 3:**
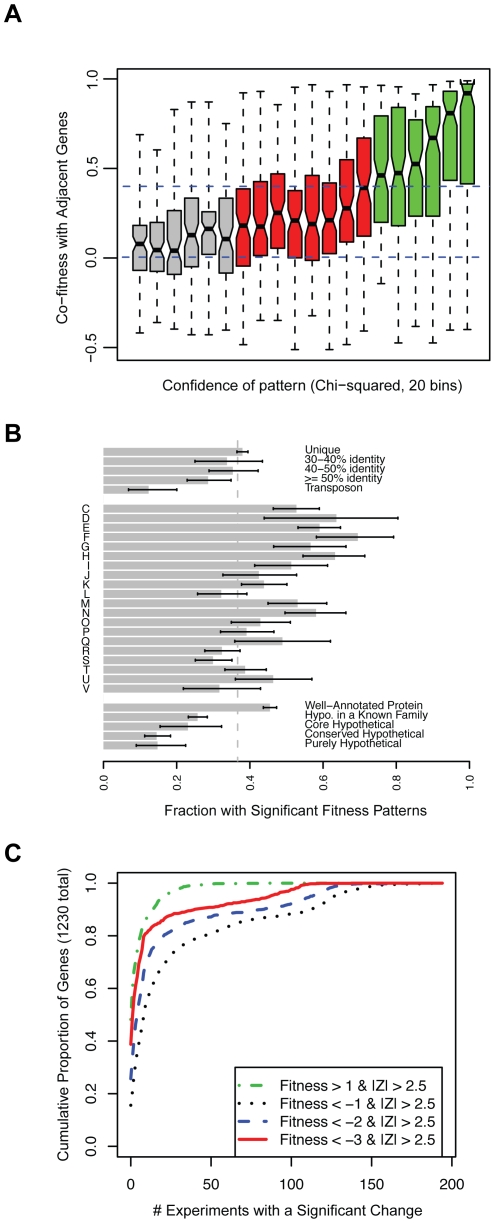
A phenotype for more than 2,000 genes in *S. oneidensis* MR-1. (A) Genes with more significant fitness patterns (higher chi-squared) tend to have stronger correlations with other genes in the same operon. We divided the genes into 20 bins by the significance (chi-squared) of their fitness patterns and for each bin we show a box plot of the correlations of those genes with adjacent genes that are predicted to be co-transcribed. The box shows the median and the interquartile range; the whiskers show the extreme values; and the indentations show the 90% confidence interval of the median. Red and green bins have statistically significant chi-squared scores (P<0.001). Dashed lines are at 0 (random) and 0.4 (highly significant cofitness; P<1e-8). (B) The proportion of different kinds of genes that have strong fitness patterns (N = 1,230; see main text). The single letter codes are COG function codes: C (Energy production and conversion), D (Cell cycle control, cell division, chromosome partitioning), E (Amino acid transport and metabolism), F (Nucleotide transport and metabolism), G (Carbohydrate transport and metabolism), H (Coenzyme transport and metabolism), I (Lipid transport and metabolism), J (Translation, ribosomal structure and biogenesis), K (Transcription), L (Replication, recombination and repair), M (Cell wall/membrane/envelope biogenesis), N (Cell motility), O (Posttranslational modification, protein turnover, chaperones), P (Inorganic ion transport and metabolism), Q (Secondary metabolites biosynthesis, transport and catabolism), R (General function prediction only), S (Function unknown), T (Signal transduction mechanisms), U (Intracellular trafficking, secretion, and vesicular transport), and V (Defense mechanisms). Unique genes (top of panel) are those without a homolog in the MR-1 genome at greater than 30% amino acid identity. The error bars are 90% confidence intervals. (C) The cumulative proportion of all genes with strong fitness patterns (N = 1,230) versus the number of experiments with a significant change. Here we define a significant change as |Fitness|>1 and |Z|>2.5. Four classes of significant change are plotted; fitness defects of varying severity or positive fitness. For example, ∼40% of the 1,230 genes do not have a severe fitness defect (Fitness<−3) in any of the 195 conditions despite having a strong fitness pattern and 80% of genes with a severe fitness defect (Fitness<−3) have that phenotype in 10 experiments or less.

Nevertheless, many of these fitness patterns are weak and it is difficult to place these genes into pathways because the phenotypes are too subtle. To identify those genes with a strong fitness pattern, we used two approaches. First, we used an arbitrary cutoff and selected genes within the top one-third of chi-squared values as having strong fitness patterns. As shown in Figure 3A, these genes with higher chi-squared values tend to show greater cofitness (correlation of fitness) with other genes in their operons. Second, as the chi-squared test may miss genes that have a fitness defect in only one condition, we also considered genes with highly significant defects in one condition (P<0.01 after Bonferonni correction, corresponding to Z<−3.88). Together, these two tests gave us 1,230 genes with strong patterns, and the remainder of the section will focus on these genes.

We next explored the properties of these 1,230 genes with a strong fitness pattern. When examined in the context of COG function codes [32], all classes of genes have significant phenotypes (Figure 3B). However, genes in characterized COG families are rather more likely to have a phenotype than genes with only general predictions or unknown roles (code R or S in Figure 3B). In addition, genes involved in “replication, recombination, and repair” (code L) are also less likely to have a phenotype, which may reflect the choice of conditions that we profiled or genetic buffering (redundancy) in these pathways. To address redundancy in a more systematic way across our entire dataset, we asked whether genes that are similar to other genes in MR-1 (paralogs) have phenotypes. We find that genes with a highly similar paralog (over 50% amino acid identity) are slightly less likely to have a phenotype than unique genes (Figure 3B). This suggests that paralogs often function under specific conditions and therefore do not always provide functional redundancy. This finding supports recent observations that most c-type cytochromes in MR-1, despite frequent sequence similarity to one another, have a detectable phenotype when deleted [33]. Lastly, “core” hypothetical genes (without a known family) that are conserved across diverse *Shewanella* genomes are more likely to have a phenotype than other hypotheticals (Figure 3B). This suggests that poorly characterized core genes, which partly define what it means to be in the *Shewanella* genus [34], are functionally more important than other poorly characterized genes.

For the 1,230 genes with a strong fitness pattern, we determined how many experiments resulted in a significant phenotype. We find that most of these genes have a clear phenotype in a small number of experiments (Figure 3C). The median of these genes has a fitness defect in 9 experiments and positive fitness in 1 experiment. By contrast, some genes have strong fitness patterns in a large number of experiments. One example is the genes of the general secretory pathway, as illustrated in Figure 1B. The individual general secretory pathway genes have a complex, highly correlated fitness pattern and their absence leads to strong fitness defects or positive fitness in the majority of tested conditions.

### Evidence-based annotation of gene function using fitness profiles

One of the main aims of this study was to use mutant fitness to annotate gene function. Using the approaches described below, we predicted specific functional annotations for 40 genes/operons with either poor or incomplete annotations including 17 enzymes, 10 transporters/efflux pumps, 4 transcriptional regulators/signaling proteins, 4 electron transport proteins, and 5 other proteins (Table 1 for list; Text S2 for detailed rationale). Even in retrospect, few of these annotations could have been made by homology alone. First, we identified those genes with a strong fitness defect in only one or a few conditions, as an annotation of a specific function in these instances is often easier than if a gene has pleiotropic effects. Second, we identified groups of genes with high cofitness across the entire compendium. These genes are more likely to be functionally related and allow for functional annotation if one or more of the genes in the cofitness cluster are characterized [7]. Given these genes and their phenotypes, we used comparative genomics and prior experimental data from MR-1 and other bacteria to predict specific functions. We split our evidence-based annotations into three categories, “new”, “expanded”, and “confirmed”, to reflect prior knowledge and genes that have multiple functions. Selected examples of evidence-based annotations and their experimental validation are described below.

**Figure 4:**
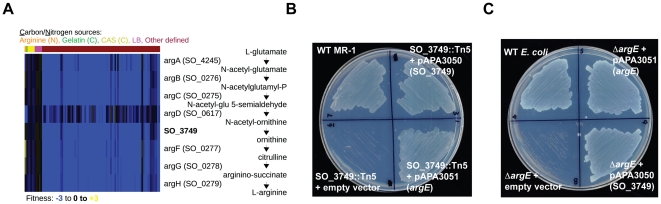
SO_3749 encodes a functional N-acetyl-ornithine deacetylase. (A) Fitness heatmap for genes of the arginine biosynthesis pathway and SO_3749, annotated as a hypothetical protein. (B) Growth of wild-type MR-1 and a SO_3749 transposon mutant on minimal media with DL-lactate as a carbon source. The auxotrophy of the SO_3749 mutant is complemented by the *E. coli argE* gene (bottom right). (C) Same as (B) for wild-type *E. coli* and an *argE* deletion mutant. The auxotrophy of the *E. coli argE* mutant is complemented by MR-1 SO_3749 (bottom right).

Some of our gene annotations reflect specific new functions for hypothetical genes. For example, the MR-1 genome does not have an annotated enzyme for synthesizing ornithine from N-acetyl-ornithine, a necessary step in arginine biosynthesis. Given that MR-1 is capable of synthesizing arginine, we sought to identify the missing enzyme. The N-acetyl-ornithine to ornithine reaction is encoded in *E. coli* by *argE*, an N-acetyl-ornithine deacetylase, and in *B. subtilis* by *argJ*, an ornithine acetyltransferase. Given that MR-1 does not have homologs to either *argE* or *argJ*, we examined our fitness data for uncharacterized genes with high cofitness with known arginine biosynthesis genes to identify the missing enzyme. Using this approach, we identified high cofitness between mutants in the hypothetical gene SO_3749 and mutants in other arginine biosynthesis genes (Figure 4A). The outlier in Figure 4A, *argD*, participates in other metabolic processes including lysine biosynthesis that may contribute to its complicated fitness pattern. SO_3749 contains a hydrolase domain, so we predict that its activity resembles that of ArgE rather than the transacetylase ArgJ. To verify that SO_3749 encodes the missing step in arginine biosynthesis, we performed cross-species complementation assays. First, we complemented the minimal media growth deficiency of a MR-1 SO_3749 transposon mutant with the *E. coli argE* gene (Figure 4B). In addition, we complemented the growth defect of an *E. coli argE* deletion strain with a plasmid expressing the SO_3749 gene (Figure 4C). Despite lacking any detectable homology to *E. coli argE*, our results demonstrate that the hypothetical gene SO_3749 encodes a functional N-acetyl-ornithine deacetylase (or ornithine acetyltransferase).

**Figure 5:**
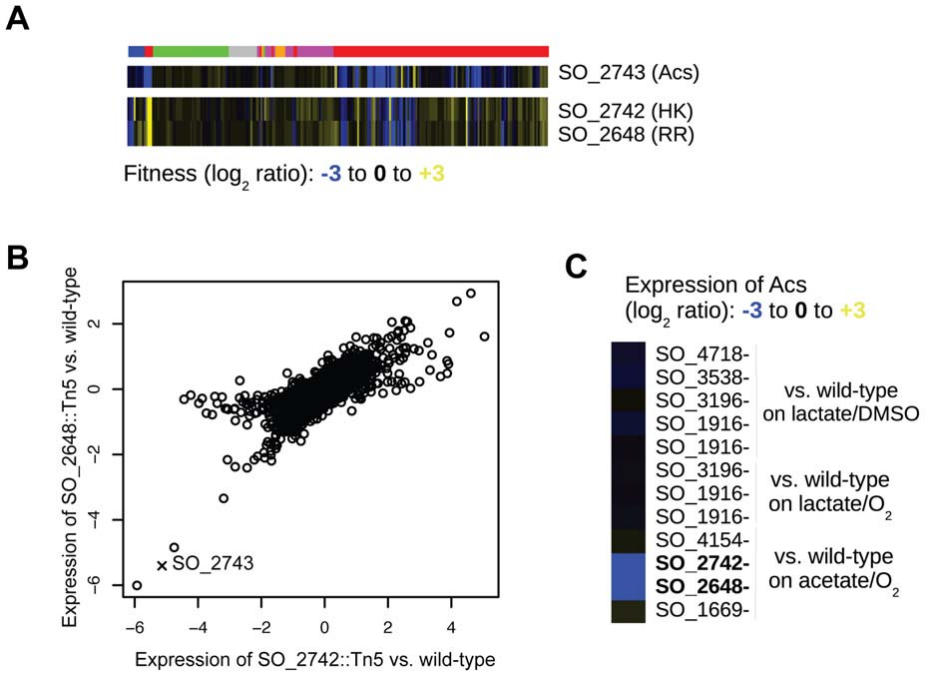
SO_2648 and SO_2742 activate *acs*. (A) Fitness pattern of histidine kinase SO_2742, response regulator SO_2648, and acetyl-coA synthetase (*acs*). SO_2742 and SO_2648 have highly correlated fitness patterns over the entire compendium. The color code for experiments is identical to that in Figure 1B. (B) Comparison of genome-wide expression in mutants of SO_2648 and SO_2742. RNA samples for both mutants and wild-type were collected one hour after transfer to a minimal media with acetate as the carbon source. The expression of both mutants is plotted as the log_2_ ratio of mutant versus wild-type. The expression of *acs* is marked with an X. (C) Relative expression of *acs* in different transcription factor mutants and conditions. For each mutant, the expression level is relative to wild-type MR-1 grown in the identical condition. Lactate/DMSO is one hour after transfer to anaerobic minimal media with DL-lactate as a carbon source and DMSO as an electron acceptor, acetate/O_2_ is one hour after transfer to aerobic minimal media with acetate as a carbon source, and lactate/O_2_ is aerobic exponential growth in DL-lactate minimal media.

We next used our fitness compendium to uncover new two-component signal transduction pathways. In MR-1, as in many bacteria, the majority of histidine kinases (HK) and their cognate response regulators (RR) are cotranscribed in operons [35]. However, there are often instances of “orphan” HKs and RRs in the genome for which the cognate partners are unknown. Using cofitness as a metric for functional interactions, we find that two orphan HK-RR pairs have highly correlated fitness. In the first example, hybrid HK SO_3457 and RR SO_1860 have high cofitness (r = 0.83; Pearson correlation) and are required for motility, growth in high and low pH, and growth on a large number of carbon and nitrogen substrates. The *E. coli* orthologs of these genes, *barA* and *uvrY*, form a two-component system that ultimately regulates the global regulator CsrA [36]. Gene expression experiments with SO_1860 and SO_3457 mutant strains suggest that CsrA is not a target of this two-component system in *Shewanella* (data not shown) demonstrating that this system may regulate different genes. There inferences were confirmed by a very recent report on *barA* and *uvrY* in MR-1 [37]. In a second example, we find that HK SO_2742 and RR SO_2648 have high cofitness (r = 0.85; Pearson correlation) and are required for optimal growth in minimal media with acetate, propionate, or butyrate as carbon sources (Figure 5A). To further demonstrate a functional relationship between SO_2742 and SO_2648 and to look for target genes of the RR, we assayed transcript levels in mutants of both the HK and RR after transfer to minimal media with acetate as a carbon source. We found that the expression of genes in these mutants is highly correlated further suggesting a direct biochemical interaction between the HK and RR (Figure 5B). Lastly, we identify *acs* (SO_2743; encoding acetyl-coA synthetase), which is divergently transcribed from the adjacent SO_2742, as significantly downregulated in both mutants (Figure 5C). In *E. coli*, *acs* activates acetate to acetyl-CoA and an *acs* mutant grows poorly in acetate containing media [38]. Based on this data, we propose that the MR-1 two-component system SO_2742 and SO_2648 senses carboxylates and that one of its primary targets is *acs*.

A combination of mutant fitness profiles and comparative genomics is a powerful method for predicting gene function. For instance, mutants in the hypothetical gene SO_1913 have high co-fitness with mutants in the general secretory pathway (Figure 1B). To infer the potential role of SO_1913 in the general secretory pathway, we looked for previous experimental evidence from homologs in other species. Using this approach, we identified a positional ortholog in *Shewanella benthica* KT99 (KT99_05357) that is similar to the type III secretion chaperone *yscW* (PF09619) [39]. Additionally, we found that some homologs of SO_1913 in other species are fused with an uncharacterized meta/hslJ domain (for example, Lferr_0115 from *Acidithiobacillus ferrooxidans* ATCC 53993) that is predicted to be associated with heat shock (and hence chaperone) proteins. Based on our mutant fitness data and the comparative analysis, we propose that SO_1913 is a chaperone for the general secretory pathway. In a second example, we identified two conserved hypothetical genes (SO_3259-SO_3260) that are required for motility. These genes are located adjacent to a large flagellar/chemotaxis gene cluster suggesting that SO_3259-SO_3260 may play a direct role in motility. To identity a putative function for SO_3259-SO_3260, we analyzed homologs with experimental evidence in other species. We find that SO_3259 is similar to the flagellar modification genes *pseD* and *pseE* from *Campylobacter jejuni*, that are involved in decorating the flagellum with sugars and are required for full motility [40]. Therefore, we propose that the SO_3259 and SO_3260 participate in the modification of the flagellum by some sugar and that this modification is required for motility. In a final example, we find that the previously hypothetical gene SO_0444 is required for growth in ZnSO_4_ or CuCl_2_ stress conditions. SO_0444 is in an operon with and predicted to be regulated by SO_0443 [41], a putative ortholog of the *E. coli* zinc-responsive transcriptional regulator ZntR [42]. Given that SO_0444 is predicted to be a membrane protein, we propose that this previously hypothetical gene encodes a copper/zinc efflux protein.

In addition to new annotations, analysis of mutant fitness profiles can directly confirm previous predictions of gene function and also expand our current understanding of a single gene's activity. The operon SO_1427-SO_1432 encodes a DMSO reductase that functions under anaerobic conditions [43]. In addition to its essential role in DMSO reduction, we find that mutants in SO_1427-SO_1432 are also impaired in their ability to use manganese oxide as a terminal electron acceptor, thus expanding the known substrate range of this reductase. Given that MR-1 was isolated under manganese reducing conditions [21], the finding that a DMSO reductase plays a role in manganese oxide reduction is particularly interesting.

### Mutant fitness and gene expression are poorly correlated

Whole-genome gene expression profiling using microarrays is standard practice in microbiology. Typically, these studies are designed to detect differential expression between two conditions (i.e., treatment versus control or mutant versus wild-type) in an effort to identify those genes whose expression is under regulation (for example, see [44]). The expectation in these experiments is often that genes with differential expression are more likely to be functionally important (and hence have a fitness consequence when mutated). However, the extent to which this is true, that is the correlation between differential gene expression and mutant fitness, has to our knowledge not been systematically investigated in bacteria.

**Figure 6:**
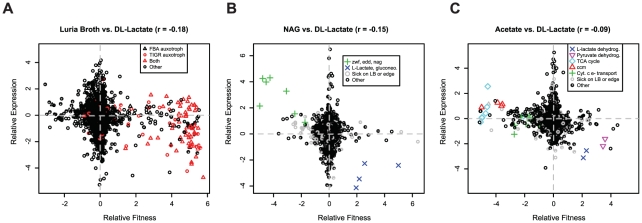
Gene expression and mutant fitness are poorly correlated. (A) Comparison of gene expression and mutant fitness in LB (rich media) versus minimal media with DL-lactate as a carbon source. Relative expression is a comparison of gene expression for wild-type MR-1 in exponential growth in the two conditions. Relative fitness is the difference of pooled fitness values for the two conditions. Both relative fitness and expression values are log_2_ ratios. For example, genes on the bottom right of the plot are up-regulated in expression in DL-lactate minimal media relative to LB and are more important for fitness in DL-lactate minimal media than in LB. Therefore, a correlation of -1 would be a perfect correlation between mutant fitness and gene expression. FBA auxotrophs are predicted from flux balance analysis [30]; TIGR auxotrophs are predicted from TIGR functional roles [29]. (B) Same as (A) for minimal media with DL-lactate and N-acetyl-glucosamine (NAG) as carbon sources. Gene codes correspond to *edd* (SO_2487; phosphogluconate dehydratase), *zwf* (SO_2489; glucose-6-phosphate 1-dehydrogenase), and nag (*nagP* (SO_3503), *nagA* (SO_3505), *nagB-II* (SO_3506), *nagK-I* (SO_3507), and *nagR* (SO_3516)). The NAG genes were annotated by Osterman and colleagues [80]. Sick on LB or edge indicates if a gene is sick on LB (which means that the gene is likely sick in many conditions) or insertions for that gene are only on the edge (not within the central 5–80% portion of the protein). (C) Same as (A) for minimal media with DL-lactate and acetate as carbon sources. Gene codes correspond to L-lactate dehydrogenase (SO_1518:SO_1519), pyruvate dehydrogenase (SO_0424:SO_0425), ccm – cytochrome c maturation (SO_0259:SO_0268), cytochrome c electron transport genes (SO_2357:SO_2364; SO_0608:SO_0610), and TCA cycle and related genes (SO_0770, SO_1483:SO_1484, SO_2339:SO_2341, SO_3855).

To address this issue we assayed transcript levels for wild-type MR-1 grown in rich media (LB) and in minimal media with DL-lactate, acetate, or N-acetyl-glucosamine (NAG) as carbon sources and compared differential expression to differential fitness values obtained from identical growth conditions. The genome-wide correlation between mutant fitness and gene expression, while statistically significant, is weak (Figure 6). The only genes that “make sense”, which we define as those that are upregulated in condition A relative to condition B and also important for fitness in condition A relative to condition B, are a few key genes that directly contribute to the utilization of the carbon substrate. For instance, genes of the NAG utilization operon are important for fitness and are upregulated in NAG-containing media (Figure 6B). However, in every comparison, there are genes with fitness defects when mutated in a single condition whose expression is not differentially regulated under the same condition. Conversely, in all comparisons there are many genes that are differentially expressed but have no fitness consequence. Our findings are similar to those in yeast where the correlation between mutant fitness and gene expression is also weak [8], [45]. Therefore, the lack of correlation between gene expression and mutant fitness is a general trend. Theories that might explain the lack of correlation include standby expression [46], anticipatory control [47], [48], suboptimal control of recently acquired genes [49], or post-transcriptional control.

### Comparison of information content of mutant fitness and gene expression

Given the extensive use of gene expression profiling in bacteria and the relative lack of large-scale mutant fitness studies, it is important to examine the information content of both assays. In particular, for a single organism, we compared the ability of large expression and mutant fitness datasets to predict gene function and gene regulation. For the mutant fitness dataset, we used the data from the 195 experiments described in this paper. For gene expression, we used the data from 371 experiments contained in the MicrobesOnline website [50]. These gene expression experiments are derived from this study and others (for example; [51], [52]) and represent a diverse range of conditions comparable to what we describe here for mutant fitness.

**Figure 7:**
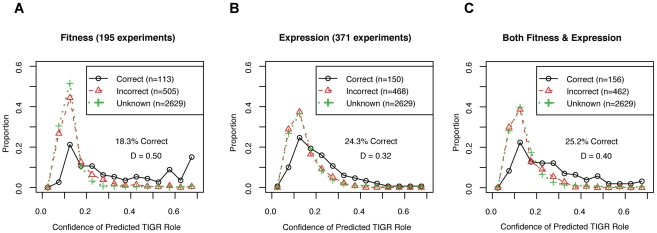
Mutant fitness gives confident predictions of gene function. (A) We used 195 fitness experiments to predict TIGR functional groups or subroles. We show the distribution of confidence values for correct and incorrect predictions for the 618 genes with subroles assigned by TIGR and also the distribution of confidence values for the 2,629 genes that were not assigned subroles by TIGR (in green; marked as unknown). The confidence of correct predictions is significantly better than for the incorrect predictions (Kolmogorov-Smirnov test; D = 0.50, P-value<1e^−15^). (B) Same as (A) but using 371 microarray gene expression experiments. (C) Same as (A) but using both 195 pooled fitness experiments and 371 gene expression experiments.

For gene function predictions, we tested the ability of the large-scale expression and/or mutant fitness datasets to place genes into pathways. Of 3,247 genes that were in both datasets, the TIGRFam database assigned 618 genes to 79 different “subroles” such as “Amino acid synthesis: Aspartate family” by homology [29]. To classify the genes into subroles from the data, we used a standard machine learning technique called random forests that is resistant to overfitting and that estimates the confidence of its predictions. We trained the classifier using the 618 genes that have assigned subroles, and then predicted the TIGR subroles for all 3,247 genes given the data (We used 10-fold operon-wise cross-validation to avoid overfitting the genes with known subroles, see Materials and Methods). For the 618 genes with known subroles, most of the predicted subroles did not match TIGRFam annotations, but predictions with confidence values above 50% were likely to be correct (Figure 7). We find that gene expression gives more correct gene function predictions than mutant fitness but fitness gives more high-confidence predictions (Figure 7A and 7B). Furthermore, combining the fitness and expression datasets together did not increase the confidence of the predictions based on mutant fitness alone (Figure 7C). The higher confidence of mutant fitness-based predictions confirms our intuition that mutant phentotypes give more direct information about gene function than gene expression patterns do.

**Figure 8:**
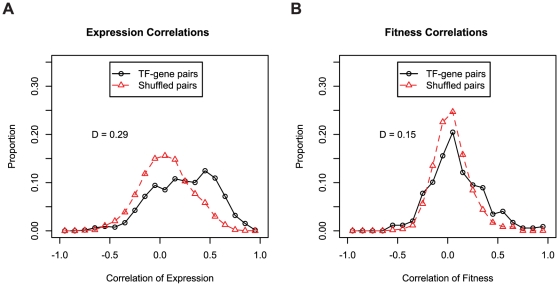
Expression and fitness are both informative about gene regulation. (A) Coexpression of transcription factors and their predicted target genes. All transcription factor-target gene pairs are derived from the manually curated RegPrecise database [41]. We excluded autoregulatory pairs from both the RegPrecise gene pairs and the shuffled controls. The same 371 expression experiments described in Figure 7B are used. (B) Same as A, using cofitness rather than coexpression. For fitness correlations, the entire fitness compendium of 195 experiments was used.

To determine the ability of gene expression and mutant fitness to predict gene regulation, we examined the expression and fitness correlation of MR-1 transcription factor-target gene pairs from the RegPrecise database [41]. We find that transcription factor-target gene pairs tend to have higher coexpression than cofitness correlations, relative to shuffled gene pairs (Figure 8). Interestingly, pairs with high correlation in one dataset tend not be highly correlated in the other (Pearson correlation r = 0.09; even this correlation disappears if co-transcribed transcription factor-gene pairs are excluded from the analysis). Thus, the two datatypes, expression and fitness, should be complementary for analyzing gene regulation. To illustrate this point, we examined our fitness dataset and identified strong cofitness (r = 0.56; Pearson correlation) between the uncharacterized transcription factor SO_1916 and its divergently transcribed neighbor gene SO_1917, a putative efflux pump. Conversely, the expression correlation between SO_1916 and SO_1917 is weak (r = 0.13; Pearson correlation). SO_1916 and SO_1917 are required for optimal growth under certain anaerobic conditions with DMSO as an electron acceptor (Figure 2E). To demonstrate that SO_1916 regulates SO_1917, we performed gene expression experiments on SO_1916 mutants after transfer to anaerobic conditions with DMSO as an electron acceptor (Figure S4). Relative to wild-type, we found a DMSO-specific down regulation of SO_1917 in two independent SO_1916 mutant strains (Figure S4). These data suggest that SO_1916 is a neighbor regulator that activates SO_1917 expression and that the activity of the efflux pump encoded by SO_1917 is necessary when DMSO is an electron acceptor.

Overall, we conclude that large-scale gene expression and mutant fitness datasets provide complementary information. Mutant fitness gives higher confidence predictions of gene function and is better suited to annotating the function of genes with a low false-positive rate (Figure 7). Conversely, the analysis of expression datasets is a better methodology for elucidating gene regulation (Figure 8). However, it is important to note that fitness and expression datasets are not limited to gene function annotation and gene regulation, respectively. Gene expression is an established method for predicting gene function [53] albeit at lower confidence than mutant fitness (Figure 7B). Conversely, as described above for SO_1916, mutant fitness can lead to regulatory insights that may be missed by expression profiling alone.

## Discussion

### Evidence-based annotation of gene function using mutant fitness

The systematic determination of gene function across the diversity of bacteria is a major challenge in microbiology. Previous studies have demonstrated the utility of high-throughput mutagenesis and phenotyping strategies to annotate bacterial gene function [10], [54]. Nevertheless, these studies were only able to assign specific functions to a small number of genes. To demonstrate the utility of mutant fitness for annotating gene function on a larger scale in a non-model bacterium, we described the construction of a near-complete set of archived mutants in *Shewanella oneidensis* MR-1 and the fitness profiling of this collection in over 100 diverse conditions. The entire fitness dataset is available on the MicrobesOnline website [50] and for download (http://genomics.lbl.gov/supplemental/MR1fitness2011/). In addition to identifying a strong phenotype pattern for 1,230 genes, of which 627 (51%) lack an *E. coli* ortholog and 282 (23%) are annotated as hypothetical, we used gene fitness profiles to derive ‘evidence-based’ gene annotations for 40 genes/operons and we verified some of these functions experimentally. On average, each reannotated gene has a tree-ortholog in 97 (5%) of 1,828 other bacterial genomes in MicrobesOnline [50]. Indeed, most bacterial genomes (73%) contain at least one tree-ortholog of these genes, which illustrates that our mutant fitness data and the evidence-based annotations are relevant to most bacteria.

### Techniques for large-scale bacterial mutant fitness profiling

The approach presented here for MR-1 uses artificial DNA tags engineered into transposons and a pooled growth assay in order to determine relative fitness of each mutant strain. DNA tags, as best exemplified in yeast and in this study, are a powerful method for generating quantitative fitness data using a simple experimental assay and sample-processing step [55]. Nevertheless, the approach presented here requires archived strains and the mutant pool size is limited to the number of unique TagModules that are available. Archiving the mutant strains is beneficial for follow-up studies, in particular when an efficient system for constructing targeted mutations, such as recombineering in *E. coli* [56], is not available. Archived strains also enable additional unpooled assays, for example studies of protein localization [57] or metabolomics [58]. However, given the number of poorly characterized microbial species and the continuing drop in sequencing costs, it is likely that future fitness datasets will be generated using pool-based approaches that do not require strain archiving such as HITS [59], TraDIS [60], Tn-seq [61], [62], and TRMR [63].

In addition to pool-based approaches described above, the high-throughput imaging of clonal mutants has also been used to generate a large-scale gene-phenotype map in bacteria, as recently demonstrated in *E. coli* [10]. To compare the two methodologies, we examined the operon fitness correlations from our tag-based MR-1 dataset and the colony size-based *E. coli* dataset. We find that the operon fitness correlations are significantly better for the MR-1 compared to the *E. coli* dataset, even for matched conditions (Figure S5). Given that polar effects should be similar in both assays (a dominant drug marker marks both the targeted *E. coli* deletions and the MR-1 transposon insertions), we conclude that the DNA tag-based assay is more quantitative and better suited for identifying small fitness defects. However, we found that some MR-1 pool experiments (for example, succinate as a carbon source) consistently gave low-quality fitness data by our metrics, presumably because a handful of strains take over the population and skew the relative fitness values (data not shown). A similar phenomenon has been observed in pooled transposon mutant studies in *E. coli* [64], suggesting that certain conditions are best assayed as single mutants rather than pool-based assays.

### Towards a phenotype for every gene in a bacterial genome

Regardless of which method is used to generate large gene-phenotype maps in bacteria, the challenges associated with this data are common. Foremost, most genes in the genome either have a weak phenotype or no phenotype at all and therefore predicting functions using mutant fitness patterns is not possible. Given that strong phenotypes are easier to assay and interpret, one pressing question is what is necessary to identify strong phenotypes for all genes in a bacterial genome? One option is to profile the single gene mutation collection under a more diverse set of laboratory conditions, including a large number of chemical inhibitors with different modes of action, as in yeast [17] and *E. coli* [10]. An alternative is to assay the mutant collection in more ecologically relevant conditions under the hypothesis that some genes may perform environment-specific functions (for instance, cell-cell communication with a different species) that are difficult to recapitulate in the laboratory. To lend credence to this hypothesis, a study looking at the survival of MR-1 mutants in sediment identified phenotypes for some genes that we failed to find a phenotype for using our more standard laboratory assays [65]. A third option is to systematically construct double mutations, similar to that described in *E. coli* [66], [67], under the hypothesis that bacterial species have functionally redundant genes and pathways. Each of the above approaches assumes that each gene actually has a functional consequence to the cell. However, a microbial genome is a snapshot in evolutionary time and some genes are under weak selection and are in the process of being lost [68]. It is unlikely that a strong phenotype, if any at all, will be found for all of these genes.

### Future directions for evidence-based annotation of bacteria

Studies such as ours are part of a systematic effort to move from sequencing bacterial genomes to understanding the function of all genes. However, mutant fitness as described in this study is only one piece of experimental evidence for predicting gene function. Furthermore, proving mutant fitness-based annotations requires additional investigation including metabolite and enzymatic assays at the level of single genes. To improve and extend gene annotations to a greater percentage of the genome, it is clear that additional pieces of evidence such as protein-protein interactions [69], [70], biochemical activity, and metabolomics [71] will be necessary to supplement the existing large-scale mutant fitness and gene expression datasets, which are currently easier to generate and are sure to proliferate in the near future. The integration of diverse data types for a single bacterium should provide insight into the function for many uncharacterized bacterial genes for which we have strong phenotypes but no functional predictions. Ultimately, the functional annotation of a greater number of sequenced microbial genes promises to aid future efforts in drug discovery, bioengineering, and biotechnology.

## Materials and Methods

### Strains and media

*S. oneidensis* MR-1 was purchased from ATCC (catalog number 700550). *E. coli* conjugation donor strain WM3064 was a gift of William Metcalf (U. of Illinois). The *E. coli* Δ*argE* strain was obtained from the KEIO collection [11]. See Table S4 for the strains used in this study. All strains were commonly cultured in Luria-Bertani broth (LB) with appropriate antibiotic selection; kanamycin (50 µg/ml) for MR-1 transposon mutants and for the *E. coli ΔargE* strain, and gentamicin (15 µg/ml) for complementation strains containing plasmid pBBR1-MCS5. To grow the diaminopimelic acid (dap) auxotroph WM3064, dap was added to the media at a final concentration of 300 µM. Our standard MR-1 minimal media contained salts (per liter: 1.5 g NH_4_Cl, 0.1 g KCl, 1.75 g NaCl, 0.61 g MgCl_2_-6H_2_0, 0.6 g NaH_2_PO_4_), 30 mM PIPES buffer, 20 mM DL-lactate, Wolfe's vitamins, and Wolfe's minerals. For anaerobic minimal media, we added one of the following electron acceptors: fumarate (30 mM), dimethyl sulfoxide (20 mM), iron (III) citrate (10 mM), manganese (IV) oxide (30 mM), trimethylamine N-oxide (10 mM), nitrate (5 mM), or cobalt (III)-EDTA (5 mM). For anaerobic experiments, manganese oxide [72] and cobalt (III)-EDTA [73] were prepared as described. For pool experiments with alternative nutrient sources, we replaced the DL-lactate with a different carbon source, the NH_4_Cl with a different nitrogen source, both with a single carbon/nitrogen source, or the NaH_2_PO_4_ with an alternative phosphorous source. For alternative sulfur sources, we replaced all sulfate containing minerals in the Wolfe's mineral mixture with non-sulfur containing versions such that the added sulfur source served as the sole source. MR-1 was typically grown at 30°C; *E. coli* was grown at 37°C.

### Transposon mutagenesis

We previously reported the generation and preliminary analysis of a library of 7,387 transposon insertion mutants in MR-1 [19]. To achieve greater coverage of the genome, we mapped an additional 17,301 mutants using a two-step arbitrary PCR and sequencing protocol, as described [19]. Briefly, each TagModule contains two unique 20 bp DNA sequences, the uptag and downtag, each flanked by common PCR priming sites. The TagModules are cloned into a Gateway entry vector and can be readily transferred to any Gateway compatible destination vector via the LR clonase reaction (Invitrogen). We transferred the TagModules into two different transposon vectors that are active in MR-1, the *Tn*5-based pRL27 [74] and *mariner*-based pMiniHimar_RB1 [75]. The tagged transposons were introduced into MR-1 by conjugation with an *E. coli* WM3064 donor strain carrying the appropriate suicide vector. We used a two-step arbitrary PCR and sequencing protocol to simultaneously map the transposon insertion location and identify the TagModule. All mutants were stored as glycerol stocks in either 96-well or 384-well plates. Overall, we observe transposon insertion biases both on the main 5 MB chromosome and on the 161 kB megaplasmid (Figure S6). Analysis of the insertion preferences for *Tn*5 and *mariner* indicates that their insertion biases are not equal (Figure S7), thus illustrating the benefit of using multiple transposons to achieve maximal coverage. Additionally, we observed a greater than 4-fold increase in mapped megaplasmid insertions relative to the expectation (based on size). Given that the megaplasmid appears to be equal in copy number to the main chromosome (data not shown), we speculate that the megaplasmid is more accessible to transposon mutagenesis. The total MR-1 transposon collection consists of 24,688 archived strains and represents mutants in 3,447 unique genes (Table S1 for full list).

### Pool fitness assays

We constructed two mutant pools, the upPool and dnPool, by mixing equal volumes of overnight LB cultures for each strain. These pools were designed such that each strain's TagModule is unique within that pool and to achieve maximal coverage of the genome. The upPool contains 4,058 strains whose tags are detected at a threshold ∼5× over background in representative start hybridizations. Conversely, the dnPool has 3,977 strains that meet the same detection criteria. A total of 165 strains across both pools (∼2% of the overall number of strains that we attempted to pool) were not detected at 5× over background and fitness values were not calculated for these strains. The 2% undetected strains are primarily due to sample tracking errors, slow growth of the mutant strain during the process of pool construction, and mutations in the TagModule (data not shown). Taking into account only those strains that are detectable, our two pools contain 5,680 unique transposon mutants (2,420 mutant strains are in both pools) and represent transposon insertions in 3,345 unique protein-coding genes. For 1,675 genes, two or more independent mutants are contained in the pools (Figure S1). For most pooled fitness experiments, we performed a single experiment on each of the upPool and dnPool.

Individual aliquots of each pool were frozen at −80°C in glycerol (10% v/v). Prior to initiating a pool experiment, a single freezer aliquot of each pool was grown in LB aerobically at 30°C to mid log phase (OD_600_ = ∼2.0). At this point, we collected a sample (∼1×10^9^ cells) that we term the “start”. The start sample represents the time 0 of the experiment and is the control experiment that we compare all of our growth conditions to. The same recovered cells were typically used to inoculate the “condition” media at a starting OD_600_ of 0.01 or 0.02. For standard liquid media conditions, we typically collected condition samples after the cultures reached saturated growth, representing between 3 and 9 population doublings. Some conditions, such as LB, reach a high density and have more population doublings than certain minimal media conditions, such as butyrate as the sole source of carbon.

Liquid growth pool experiments were done in a number of formats. Aerobic minimal media experiments were done in 10 mL volumes with shaking at 200 rpm. Anaerobic experiments were conducted in hungate tubes with shaking at 200 rpm. Anaerobic experiments were set up in an anaerobic chamber (Coy) with a gas mix of 5% H_2_, 10% CO_2_, and 85% N_2_. Certain stress experiments in LB were performed in 1 mL volumes in the wells of a 24-well microplate. For these experiments, the microplate was grown in a Tecan Infinite F200 reader to measure the amount of growth inhibition caused by the stress. Our target stress concentration resulted in a ∼50% reduction of the growth rate.

For swimming motility experiments, we pipetted ∼1×10^8^ cells from the start culture into the matrix of an LB soft agar plate (0.25% w/v agar) and incubated the plate at 30°C. After 1 or 2 days, we removed cells from the outer ring (i.e. the motile cells) using a sterile razor.

For heat shock survival experiments, we incubated the aliquots of the start cells in a 42°C water bath for different amounts of time. After incubation, we used some of the cells for measuring viability by serial dilution and plating on LB. The remainder of the cells was used to inoculate a fresh tube of LB that was grown overnight (to avoid the possibility of detecting tags from dead cells). The tags from the overnight sample were hybridized and used as a measure of cell survival after heat shock. For cold survival experiments, we used the same method as for heat shock except we incubated the cells at 4°C rather than 42°C. We followed a similar method for stationary phase survival. However, in this instance, we left the pools in LB at 30°C for days after the cultures reached saturation. Again, we used some of the cells for determining viability by plating on LB plates; additional cells were used to inoculate fresh LB media. The overnight growth of these fresh cultures was used for tag array hybridization and serve as a measure of mutant survival in stationary phase. Further details on the media used and the growth conditions for each of the 195 pool experiments are contained in Table S3. Plots showing the survival of MR-1 cells after heat shock, cold incubation, and stationary phase are contained in Figure S8.

### Pool sample processing and tag microarray hybridization

Genomic DNA was isolated from each sample using either the DNeasy blood tissue kit with optional RNase treatment (Qiagen) or with a QIAxtractor genomic DNA robot (Qiagen). Approximately 100 ng of genomic DNA was used as a template to amplify the uptags from the upPool samples and the downtags from the dnPool samples using previously described primers and PCR conditions [55]. We combined uptag and downtag PCR products (10 µL of each) and hybridized to a single GenFlex 16K_v2 microarray (Affymetrix) that contains the tag complement sequences. Microarrays were hybridized, washed, labeled, and scanned as described [55].

### Tag array data analysis

Using Affymetrix .CEL files as a starting point, we first averaged the log_2_ intensities across the 5 replicate probes for each tag to obtain values for each uptag and downtag. We computed the difference (the log ratio) between these values for the condition array and the start array, to give a fitness value for each strain. Sometimes we used an average of start arrays from other experiments instead of hybridizing a start array from that actual experiment; log-levels in these independent start experiments were highly correlated (r> = 0.95). We removed strains with the lowest 2% of levels in this average start array from the analysis. We normalized the fitness values for the strains so that the median fitness for each pool and for each chromosome (the main chromosome and the megaplasmid) was zero. After this normalization, some of our experiments showed significant effects based on which 96-well plate the strain had been grown in while we were preparing our pools. So, for all experiments, we also set the median fitness of each of these groups of 96 strains to zero. See Table S5 for all strain fitness data. We computed fitness values for each gene by averaging the fitness values for all of the insertions in that gene. If a gene had one or more “good” insertions (an insertion within the central 5–80% portion of the gene), then we used only those good insertions to compute the average. See Table S6 for all gene fitness data.

To estimate the reliability of each fitness value, we took advantage of our 14 control experiments (measurements of the start pools after independent recoveries from the freezer) and the fact that we have more than one fitness measurement for most genes (i.e., more than one strain, or the single strain for the gene is in both pools). We used an approach similar to that of Efron *et al*. [76]. We first computed a t-like test statistic, which was:
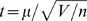


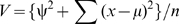
where x are the measurement(s) for the gene, μ is their average, n is the number of measurements, and Ψ = median(STD(x)), that is, the median across all genes with more than one measurement of the standard deviation of that gene's measurements. We transformed our test statistic into Z scores that follow a normal distribution in the absence of biological signal by using the control experiments; we transformed the distribution separately for genes with 1, 2, 3, or > = 4 measurements. We used conditions that we had repeated to verify that these Z scores were appropriate (i.e., the rate of discordant outliers with high |Z| values was about the same as expected by chance; data not shown). See Table S7 for Z score data.

### Individual strain growth assays

Aerobic single strain growth assays were performed in 96-well microplates in a Tecan Sunrise plate reader at 30°C with readings every 15 minutes. Anaerobic single strain growth assays were performed in a DTX880 plate reader (Beckman) housed in an anaerobic chamber (Coy) at 30°C with readings every 30 minutes. All microplate growth assays contained 150 µL per well at a starting OD_600_ of 0.02. Before all single strain growth assays, we isolated a single colony of the mutant strain and confirmed the expected location of the transposon insertion by PCR with a transposon specific primer and a genome primer. We calculated doubling times for growth curves using a logistic algorithm implemented in R. To calculate the relative growth rate for transposon mutants in Figure 1C, we divided the doubling time of the transposon mutant by the doubling time of wild-type MR-1. All single strain growth assays were performed a minimum of three times.

### Gene expression studies

We measured gene expression in wild-type MR-1 and in single transposon mutant strains. Cells were harvested after RNAprotect treatment (Qiagen) in either early exponential growth or one hour after transfer to an experimental media. For the transfer experiments, all cultures were initially cultured in DL-lactate minimal media to early exponential phase prior to transfer to the experimental media. For all experiments, we collected ∼2×10^9^ cells, isolated total RNA with a RNeasy mini kit (Qiagen), and synthesized Alexa Fluor 555 labeled cDNA with the SuperScript Plus Indirect cDNA Labeling Module (Invitrogen).

Labeled cDNA was hybridized to custom Nimblegen oligonucleotide microarrays according to the manufacturer's instructions. We used two gene expression microarray designs, a 4-plex microarray that allows hybridizing 4 samples to different regions of a single slide, and also a 12-plex microarray. The 4-plex microarray had 66,228 probes, which reduced to 61,178 after removing potential cross-hybridizing probes with BLAT, or roughly 15 probes per gene. The 12-plex microarray had 42,598 probes, which reduced to 40,881 after removal of cross-hybridizing probes. After removing potential cross-hybridizing probes, we made the distribution of the condition match that of the control (i.e., using quantile normalization), used local regression (lowess) to eliminate any bias in the log_2_ ratio by probe intensity, and set the median normalized log_2_ levels of the probes to zero (separately for each scaffold). The final log_2_ ratio for each gene was the average of these normalized values for its probes; we removed values for genes with less than 4 measurements. These data are available on MicrobesOnline. To verify the quality of each experiment, we checked the correlation of log ratios between adjacent genes that are predicted to be in the same operon and the average absolute difference within these pairs. Most comparisons had operon correlations above 0.8; some experiments had lower correlations but also had low average differences, indicating that the correlation was low because there was less biological signal to detect.

### Complementation studies

To complement the mutant phenotypes of single transposon mutant strains, we introduced an intact copy of the mutated gene on the broad-range plasmid pBBR1MCS5, which contains a gentamicin resistance marker [77]. If possible, we used the native promoter of the gene to drive expression. Otherwise, we relied on the activity of the pBBR1MCS5 *lac* promoter to express the complementation gene. The complementation plasmids were constructed with circular polymerase extension cloning (CPEC) [78] using primers 5′-GCTCTAGAACTAGTGGATCCCCC (N) and 5′- GATATCGAATTCCTGCAGCCC (N) where (N) represents genome-specific primer sequences used to amplify the gene from genomic DNA. The underlined regions represent sequences homologous to the pBBR1MCS5 backbone. To prepare the vector for CPEC, we amplified pBBR1MCS5 with primers 5′-GGGCTGCAGGAATTCGATATC and 5′- GGGGGATCCACTAGTTCTAGAGC. Following amplification, the template vector was digested with DpnI. All PCR and CPEC reactions were performed with Phusion high fidelity DNA polymerase (New England Biolabs). Both the vector and insert were gel purified with the Zymoclean Gel Recovery Kit (Zymo Research). The CPEC reaction consisted of 50 ng of linearized pBBR1MCS5, 25 ng of the complementation gene PCR product, and was cycled with the following protocol: initial denaturation for 15 seconds at 98°C, 4 cycles of 98°C for 30 seconds, 55°C for 30 seconds, and 72°C for 3.5 minutes, and a final extension at 72°C for 3 minutes. The CPEC reaction was transformed into chemically competent TOP10 cells (Invitrogen) and plated on LB with gentamicin. Complementation constructs were sequence-verified with primers comp_for_seq, comp_rev_seq, and in some instances, additional internal gene primers (see Table S8 for full list of primer sequences used in this study). Complementation plasmids were transformed into *E. coli* donor strain WM3064 and delivered into MR-1 via conjugation. Single MR-1 colonies carrying the complementation plasmids were selected on LB plates with gentamicin and assayed for growth in a microplate format as described above. See Table S9 for full list of complementation plasmids used in this study.

### Predicting TIGR subroles

TIGR roles and subroles are associated with some gene families in the TIGRFam database [29]. We obtained TIGRFam assignments from MicrobesOnline along with their associated roles and subroles. To exclude functional assignments that were not specific, we removed assignments without a subrole or that matched the strings “unknown” or “other”. This left us with 978 assignments. To predict the functional classification of genes from fitness and/or expression data, we used a standard implementation of random forests (randomForest 4.5–36 in R-2.11) and default settings. A random forest is a collection of decision trees, each of which makes their own predictions. The random forest's prediction is that predicted by the largest number of decision trees, and the confidence of the prediction is the proportion of trees in the forest that make that prediction. Before applying the random forest, we subtracted the mean from each experiment and replaced missing values with zeroes. To assess the predictions without being biased by the training data, we used 10-fold cross-validation: we trained the classifier on 90% of the genes with known subroles and then made predictions for the remaining 10% of the genes. We repeated this 10 times so that we had predictions for every gene. Because genes in the same operon tend to be functionally related, we ensured that genes in the training set (the 90%) were not in the same operons as the genes we made predictions for.

## Supporting Information

Figure S1 Coverage in MR-1 mutant pools. Coverage of protein-coding genes by the 5,680 unique strains in the two pools (upPool and dnPool). Most of the genes absent from our data (cut out portion of the pie chart) are short (<250 bp), non-unique genes such as native transposons in which insertions are difficult to map, or essential.
(PDF)

Figure S2 Fitness profiling confirms expected *S. oneidensis* MR-1 auxotrophs. Comparison of gene fitness values in DL-lactate minimal media (y-axis) and rich media (x-axis). FBA auxotrophs are predicted from flux balance analysis [30]; TIGR auxotrophs are predicted from TIGR functional roles [29]. The dashed line shows x = y.
(PDF)

Figure S3 Conservation of auxotrophs in *S. oneidensis* MR-1 and *E. coli*. We compared minimal media fitness for *E. coli* and MR-1. The *E. coli* data is from single mutant growth assays of the KEIO deletion collection in glucose minimal media [11]. The MR-1 data is from a pooled fitness assay in minimal media with DL-lactate as the carbon source. FBA auxotrophs are predicted from a flux balance analysis model for MR-1 [30]. The data are plotted for 1,086 orthologous genes for which we have data from both organisms. There is a positive fitness correlation between MR-1 and *E. coli* orthologs on minimal media (r = 0.257; Spearman correlation).
(PDF)

Figure S4 SO_1916 is a putative neighbor regulator of SO_1917. (A) Comparison of genome-wide expression in two independent mutants of SO_1916. RNA samples for both SO_1916 mutants and wild-type were collected one hour after transfer to anaerobic DL-lactate minimal media with DMSO as an electron acceptor. The expression of both mutants is plotted as the log_2_ ratio of mutant versus wild-type. The expression of SO_1917 is marked. (B) Expression of SO_1917 in different mutants and growth conditions. All expression values are log_2_ ratios of the mutant compared to the wild-type grown in the same condition. DMSO is one hour after transfer to anaerobic minimal media with DL-lactate as a carbon source and DMSO as an electron acceptor, acetate is one hour after transfer to aerobic minimal media with acetate as a carbon source, and DL-lactate is aerobic exponential growth in DL-lactate minimal media. We measured gene expression in two independent SO_1916 transposon mutants. The number/plate well listed for each gene refers to the specific transposon mutant in our collection (see Table S1).
(PDF)

Figure S5 Comparison of *E. coli* to MR-1 fitness data. Comparison of matched condition mutant fitness values for *S. oneidensis* MR-1 and *E. coli* grown in LB with either NaCl (panel A), CuCl_2_ (B), NiCl_2_ (C), or nalidixic acid (D) stress. The *E. coli* data, based on a colony size assay, is previously described [10]. For visualization, we plotted very negative values of *E. coli* fitness at −4. The plotted MR-1 stress fitness data is relative to the gene's fitness in a no stress LB culture. Points in grey have a fitness effect in LB alone (>0.5 or <−0.5, average of 5 experiments). The values are plotted for *E. coli*/MR-1 orthologs. For each condition, we note the operon correlation (opcor) for the E. coli and MR-1 datasets and the correlation (Spearman) for the fitness comparison. Only nalidixic acid has a statistically significant correlation across organisms (P = 0.00051).
(PDF)

Figure S6 *S. oneidensis* MR-1 transposon distribution. (A) Location of 21,385 transposon insertions on the main chromosome. (B) Location of 3,303 transposon insertions on the megaplasmid. (C) Increase in genes mutated as the number of mapped transposon insertions increases. All genes (n = 4,632) include all predicted protein-coding and RNA genes. Hittable genes (n = 3,820) are the subset of all genes that are unique, of sufficient length (>250 bp), and nonessential. Our final gene coverage (n = 3,447) includes 90% of the hittable genome.
(PDF)

Figure S7 Transposon insertion biases in *S. oneidensis* MR-1. (A) Distribution of insertion locations for 11,118 *Tn*5 transposon mutants on the main chromosome. (B) Same as (A) for 10,267 *mariner* transposon mutants.
(PDF)

Figure S8 Survival of *S. oneidensis* MR-1 after heat shock, cold adaptation, and stationary phase. Survival of MR-1 after heat shock at 42°C for different lengths of time. The survival plots of the upPool and dnPool are plotted separately. Cells were plated on LB plates and counted after 2 days of growth. (B) Same as (A) for cold survival at 4°C. (C) Same as (A) for stationary phase survival in LB at 30°C.
(PDF)

Table S1 List of *S. oneidensis* MR-1 transposon mutants.
(XLS)

Table S2 *S. oneidensis* MR-1 essential gene classification.
(XLS)

Table S3 List of conditions for pooled fitness experiments.
(XLS)

Table S4 List of strains used in this study.
(XLS)

Table S5 Strain fitness values 195 MR-1 fitness experiments.
(TXT)

Table S6 Gene fitness values for 195 MR-1 fitness experiments.
(XLSX)

Table S7 Z scores for 195 MR-1 fitness experiments.
(XLS)

Table S8 List of primers used in this study.
(XLS)

Table S9 List of plasmids used in this study.
(XLS)

Text S1 *Shewanella oneidensis* MR-1 essential gene analysis.
(PDF)

Text S2 Rationale for new gene annotations.
(PDF)
