# Supplementary figures and images for: Evidence-Based Annotation of Gene Function in Shewanella oneidensis MR-1 Using Genome-Wide Fitness Profiling across 121 Conditions
Source: PLoS Genet. 2011 Nov 17;7(11):e1002385. doi: 10.1371/journal.pgen.1002385 (PMC3219624; doi:10.1371/journal.pgen.1002385)

Figure S1

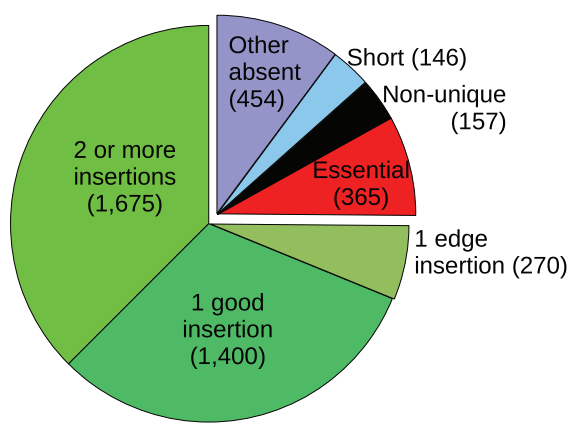

Supplement: Figure S1 — Coverage in MR-1 mutant pools. Coverage of protein-coding genes by the 5,680 unique strains in the two pools (upPool and dnPool). Most of the genes absent from our data (cut out portion of the pie chart) are short (<250 bp), non-unique genes such as native transposons in which insertions are difficult to map, or essential. (PDF) [file pgen.1002385.s001.pdf]

**Figure S2**

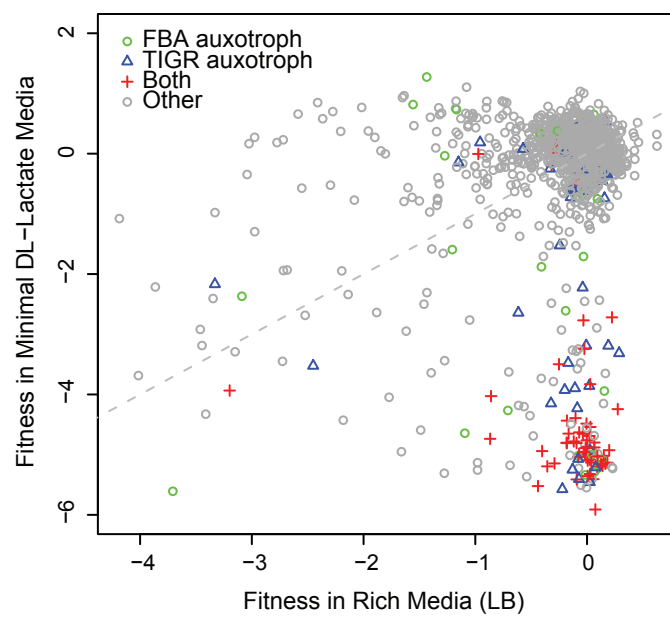

Supplement: Figure S2 — Fitness profiling confirms expected S. oneidensis MR-1 auxotrophs. Comparison of gene fitness values in DL-lactate minimal media (y-axis) and rich media (x-axis). FBA auxotrophs are predicted from flux balance analysis [30]; TIGR auxotrophs are predicted from TIGR functional roles [29]. The dashed line shows x = y. (PDF) [file pgen.1002385.s002.pdf]

**Figure S3**

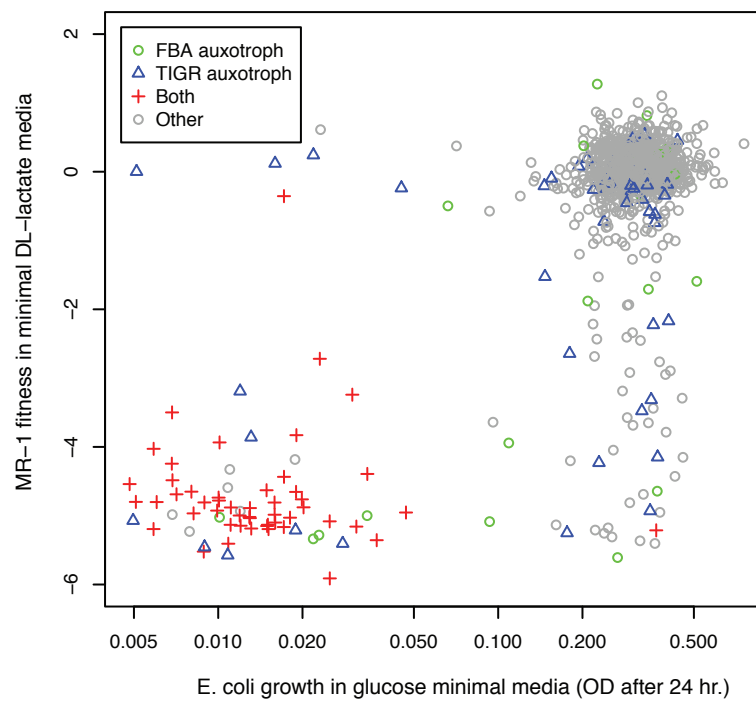

Supplement: Figure S3 — Conservation of auxotrophs in S. oneidensis MR-1 and E. coli. We compared minimal media fitness for E. coli and MR-1. The E. coli data is from single mutant growth assays of the KEIO deletion collection in glucose minimal media [11]. The MR-1 data is from a pooled fitness assay in minimal media with DL-lactate as the carbon source. FBA auxotrophs are predicted from a flux balance analysis model for MR-1 [30]. The data are plotted for 1,086 orthologous genes for which we have data from both organisms. There is a positive fitness correlation between MR-1 and E. coli orthologs on minimal media (r = 0.257; Spearman correlation). (PDF) [file pgen.1002385.s003.pdf]

Figure S4

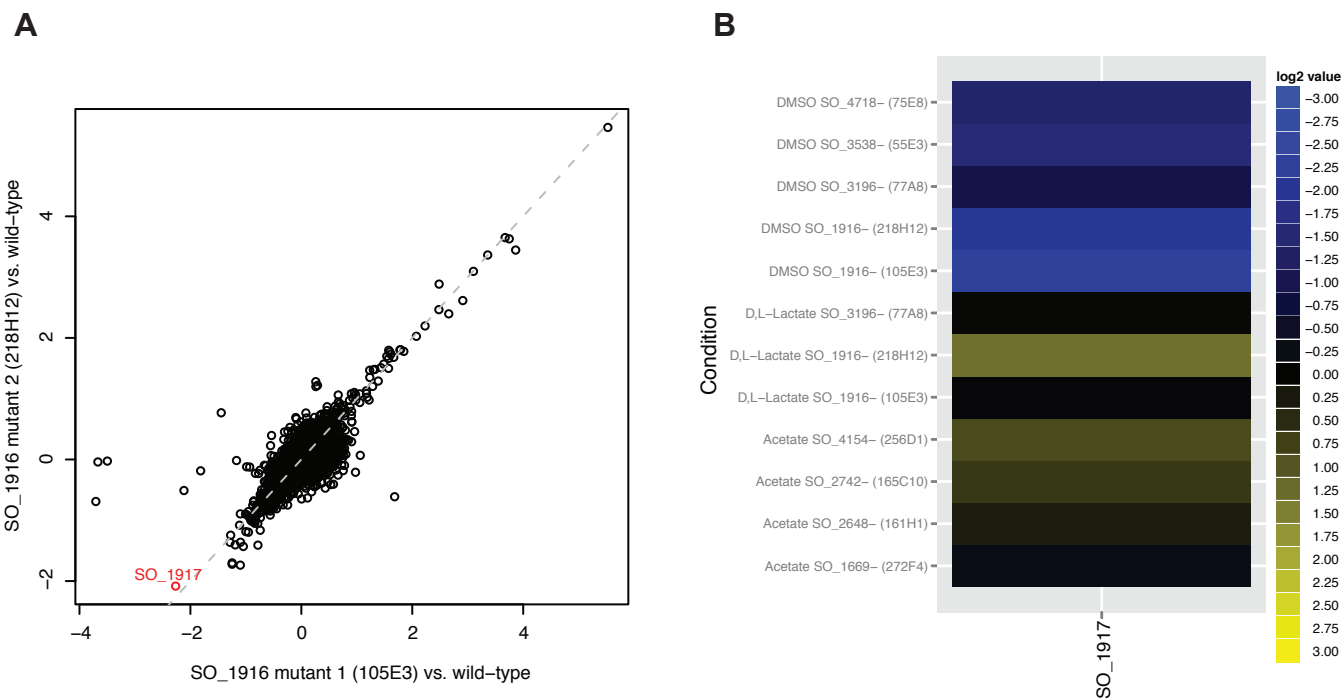

Supplement: Figure S4 — SO_1916 is a putative neighbor regulator of SO_1917. (A) Comparison of genome-wide expression in two independent mutants of SO_1916. RNA samples for both SO_1916 mutants and wild-type were collected one hour after transfer to anaerobic DL-lactate minimal media with DMSO as an electron acceptor. The expression of both mutants is plotted as the log2 ratio of mutant versus wild-type. The expression of SO_1917 is marked. (B) Expression of SO_1917 in different mutants and growth conditions. All expression values are log2 ratios of the mutant compared to the wild-type grown in the same condition. DMSO is one hour after transfer to anaerobic minimal media with DL-lactate as a carbon source and DMSO as an electron acceptor, acetate is one hour after transfer to aerobic minimal media with acetate as a carbon source, and DL-lactate is aerobic exponential growth in DL-lactate minimal media. We measured gene expression in two independent SO_1916 transposon mutants. The number/plate well listed for each gene refers to the specific transposon mutant in our collection (see Table S1). (PDF) [file pgen.1002385.s004.pdf]

Figure S5

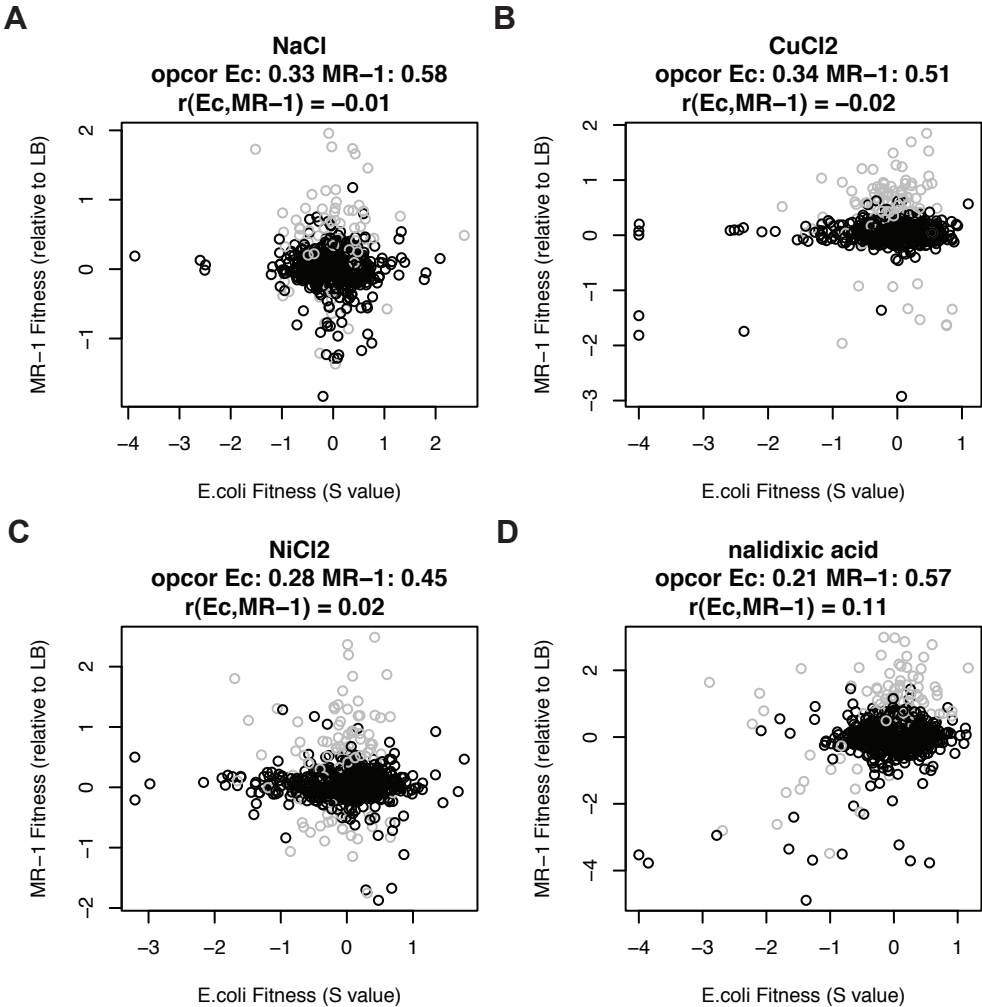

Supplement: Figure S5 — Comparison of E. coli to MR-1 fitness data. Comparison of matched condition mutant fitness values for S. oneidensis MR-1 and E. coli grown in LB with either NaCl (panel A), CuCl2 (B), NiCl2 (C), or nalidixic acid (D) stress. The E. coli data, based on a colony size assay, is previously described [10]. For visualization, we plotted very negative values of E. coli fitness at −4. The plotted MR-1 stress fitness data is relative to the gene's fitness in a no stress LB culture. Points in grey have a fitness effect in LB alone (>0.5 or <−0.5, average of 5 experiments). The values are plotted for E. coli/MR-1 orthologs. For each condition, we note the operon correlation (opcor) for the E. coli and MR-1 datasets and the correlation (Spearman) for the fitness comparison. Only nalidixic acid has a statistically significant correlation across organisms (P = 0.00051). (PDF) [file pgen.1002385.s005.pdf]

Figure S6

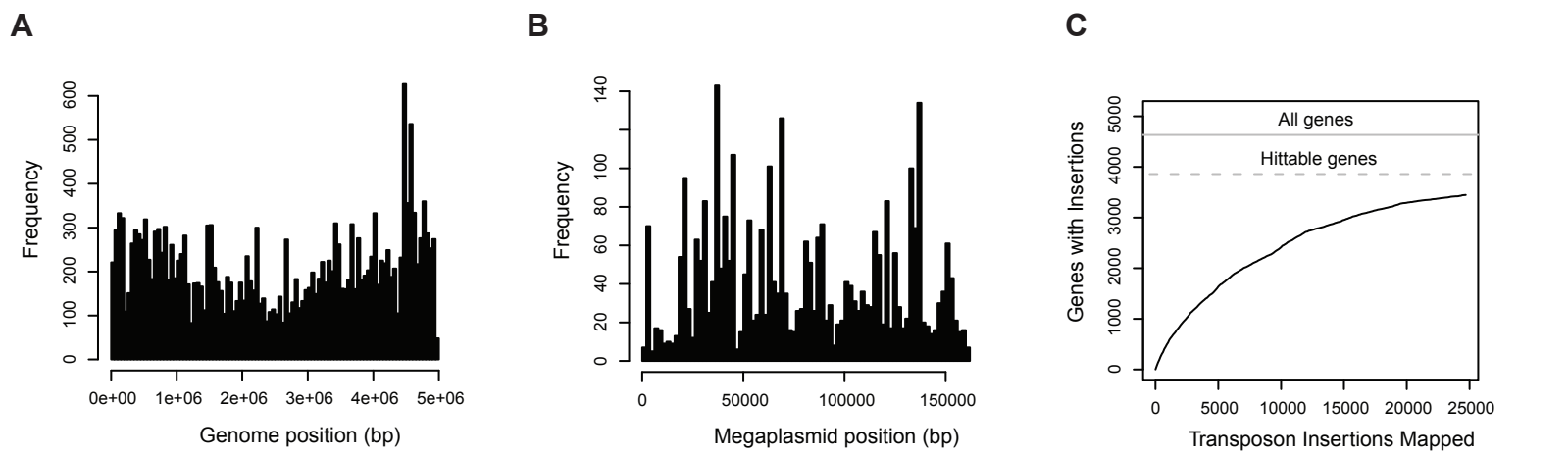

Supplement: Figure S6 — S. oneidensis MR-1 transposon distribution. (A) Location of 21,385 transposon insertions on the main chromosome. (B) Location of 3,303 transposon insertions on the megaplasmid. (C) Increase in genes mutated as the number of mapped transposon insertions increases. All genes (n = 4,632) include all predicted protein-coding and RNA genes. Hittable genes (n = 3,820) are the subset of all genes that are unique, of sufficient length (>250 bp), and nonessential. Our final gene coverage (n = 3,447) includes 90% of the hittable genome. (PDF) [file pgen.1002385.s006.pdf]

**Figure S7**

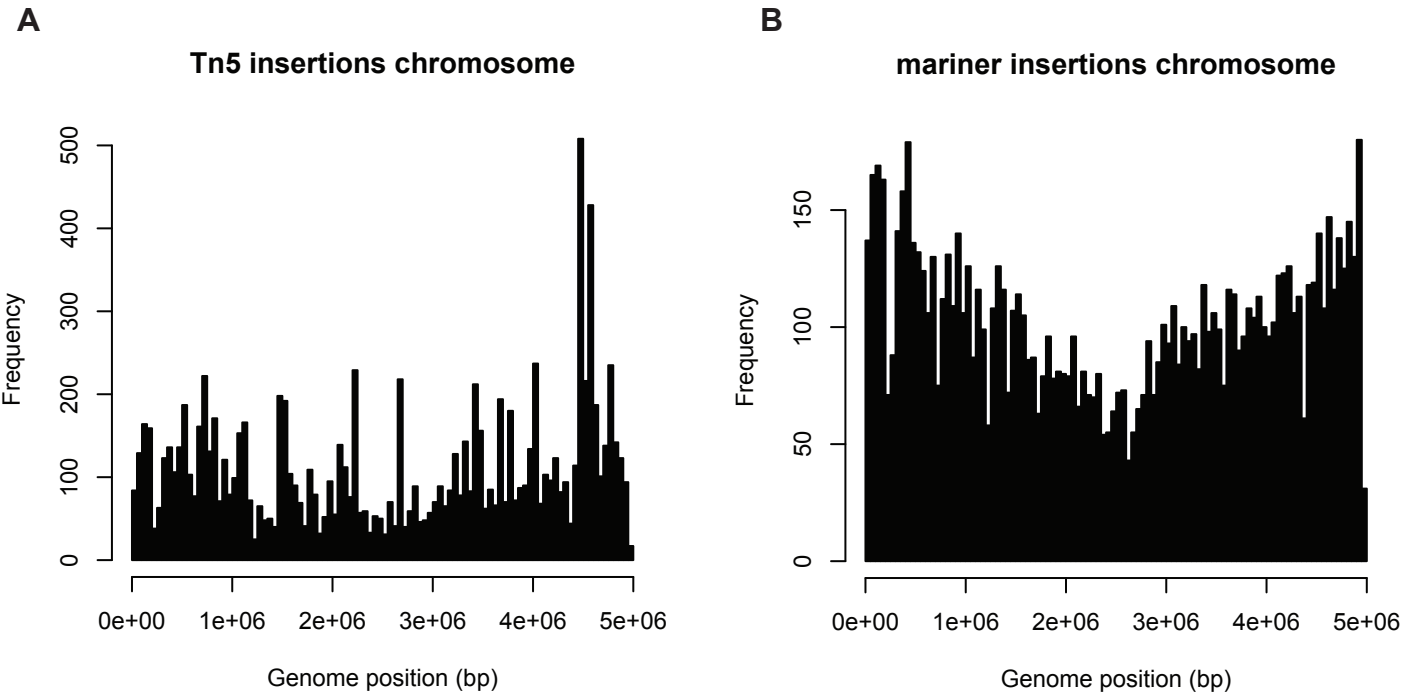

Supplement: Figure S7 — Transposon insertion biases in S. oneidensis MR-1. (A) Distribution of insertion locations for 11,118 Tn5 transposon mutants on the main chromosome. (B) Same as (A) for 10,267 mariner transposon mutants. (PDF) [file pgen.1002385.s007.pdf]

Figure S8

A

Heat shock survival at 42C

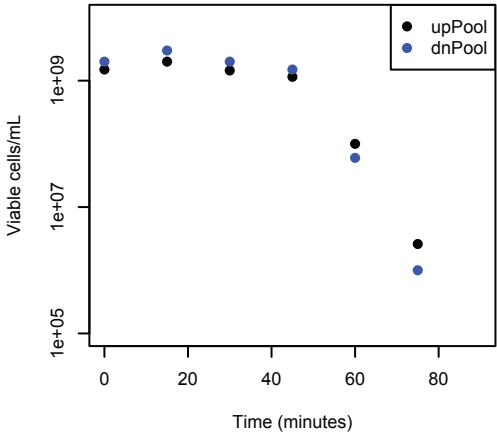

B

Cold survival at 4C

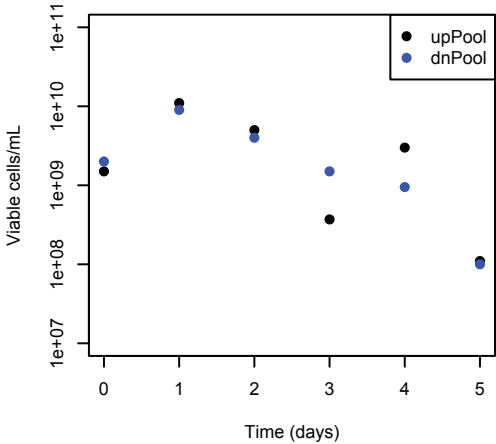

C

Stationary phase survival at 30C

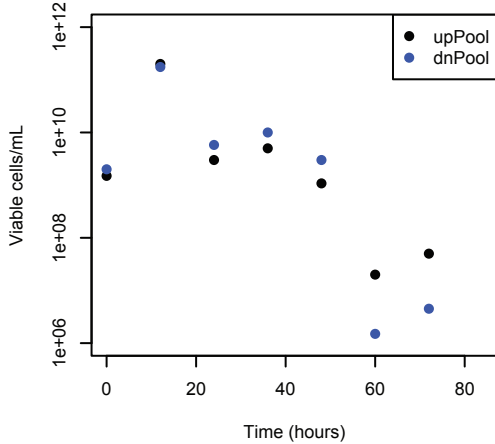

Supplement: Figure S8 — Survival of S. oneidensis MR-1 after heat shock, cold adaptation, and stationary phase. Survival of MR-1 after heat shock at 42°C for different lengths of time. The survival plots of the upPool and dnPool are plotted separately. Cells were plated on LB plates and counted after 2 days of growth. (B) Same as (A) for cold survival at 4°C. (C) Same as (A) for stationary phase survival in LB at 30°C. (PDF) [file pgen.1002385.s008.pdf]
