# Supplementary material for: Evidence-Based Annotation of Gene Function in Shewanella oneidensis MR-1 Using Genome-Wide Fitness Profiling across 121 Conditions
Source: PLoS Genet. 2011 Nov 17;7(11):e1002385. doi: 10.1371/journal.pgen.1002385 (PMC3219624; doi:10.1371/journal.pgen.1002385)
Supplement: Text S1 — Shewanella oneidensis MR-1 essential gene analysis. (PDF) [file pgen.1002385.s018.pdf]

Text S1. Essential Gene Analysis.

MR-1 genes without a transposon insertion might be essential genes in the rich media conditions used for selecting the mutants. By comparing the MR-1 transposon collection to gene essentiality data from *E. coli* [1,2] and to a lesser extent *Acinetobacter baylyi* ADP1 [3], we classified each of the predicted protein-coding genes in MR-1 into one of six essentiality classes (Table S2; see below for detailed methods). In general, MR-1 orthologs of *E. coli* essential genes were not mutated by transposon insertions in our study. We classified this group of 336 genes ExpectedEssential (Table S2) to reflect the somewhat unsurprising finding that most *E. coli* essential genes are required for viability in MR-1, a fellow γ-proteobacterium. We focused more attention on identifying those genes specifically essential in either *E. coli* or MR-1. Of particular note is the SurpiseDispensable class in Table S2, which includes genes with good transposon insertions in MR-1 whose orthologs are essential in *E. coli*. Of the 14 genes in this class, 8 (*can*, *def-1*, *hemB-1*, *hemG*, *plsC*, *rib*, *ribE-1*, *secF-2*) have paralogs in the MR-1 genome that presumably provide functional redundancy not available in *E. coli*. To identify new essential genes specific to MR-1, we identified those without good insertions (within the central 5-80% portion of the gene) that are not essential (or not present) in *E. coli* and considered the length of the gene, the local rate of transposon insertions, operon structure, whether orthologs in *Acinetobacter baylyi* ADP1 were essential, and whether the gene was conserved in other *Shewanella* species. Using these criteria, we identified 67 NewEssential genes in MR-1.

Genes putatively essential in MR-1 and not in *E. coli* reflect differences in the lifestyles of these bacteria. Unlike *E. coli*, MR-1 cannot ferment sugars and must generate energy through respiration. Consistent with this, our computational analysis identified most genes of the ATP synthase complex (*atpABDEGHI*) as essential. Additional new essential genes in MR-1 reflect differences in compound transport. Unlike *E. coli*, MR-1 is unable to uptake and utilize glucose as a substrate for growth. Consequently, we identified gluconeogenesis genes (*gpmA*, *fba*, *pgi*) as essential in MR-1. Similarly, we speculate that MR-1 is unable to transport biotin and pyridoxal phosphate (vitamin B6) from our complex (LB) media. Consistent with this notion, we identified biotin (*bioCD*) and pyridoxal phosphate synthesis genes (*pdxA*, *serC*, *pdxJ*, *pdxH*) as new (or likely) essential genes. Lastly, the ABC phosphate transport proteins SO_1560, SO1723, and SO1724 are likely essential in MR-1, which may reflect the inability of other phosphate transport systems to compensate during growth on LB. Other putatively new essential genes in MR-1 are more mysterious. We identified hypothetical genes SO4330 and SO4331 as new essentials (a neighboring gene, SO4332, also did not have a good transposon insertion). An *E. coli* homolog of SO4332, *ycaQ*, is downstream of and in a putative operon with the essential gene *lpxK*, which is required for lipid A synthesis [4]. Based on this, we speculate that SO4330-SO4332 may play an essential role in cell wall biosynthesis.

Finally, we asked whether any of the 67 NewEssential MR-1 genes are entirely new bacterial essential genes. Of the 67 NewEssential genes, 37 are essential in *Acinetobacter* ADP1. For the remaining 30, we used the DEG web site [5] to see if they were homologous to genes that are essential in other bacteria. 20 had homologs, but in a few cases, the homology was distant, and after manual inspection we decided that the genes were not actually orthologs. For example, *Haemophilus influenzae fucR* is a putatively essential ortholog of *E. coli fucR* and is quite distantly related to SO_4742, which RegPrecise [6] describes as a regulator of LPS synthesis. Overall, we have 12 truly new essential genes:

- the LPS regulator SO_4742
- the phosphate ABC transporter SO_1723:SO_1724
- stationary phase survival protein *surE* (SO_3435)
- general secretion pathway protein L (*gspL;* SO0174)
- outer membrane protein assembly complex component *bamB* (SO_3309)
- *ygfM* (SO_3310; forms complex with a periplasmic chaperone)
- *hlyD*-related transport protein SO_3278
- 23S ribosomal RNA methyltransferase *rlmN* (SO_3315)
- sugar dehydrogenase (SO_3898, possibly polar on or functionally related to *parC*)
- hypothetical proteins SO_4330:SO_4331

Methods: Essential gene analysis

The primary challenge in identifying essential genes from a random transposon study is distinguishing high-confidence new essential genes from genes that lack a transposon insertion by chance. To identify essential genes (Table S2) in MR-1, we restricted our analysis to genes on the main chromosome and used a conservative approach based on a number of factors. Foremost, we identified genes lacking a good transposon insertion, where good is defined as an insertion within the central 5-80% portion of the gene. In addition, given that our transposon collection has insertion biases (Figure S2), we calculated a Poisson P-value to ask how likely we were to see zero insertions across the genes' length, given the number of hits per nucleotide in that region of the genome. To accomplish this, we divided the genome into 50 regions of ~70 kB each with equal numbers of un-hit genes in each region. This region size was selected to give statistically significant differences across many regions. Next, we used published gene essentiality data from *Acinetobacter baylyi* ADP1 assayed in minimal media [3] and *E. coli* (two sources; a transposon footprinting experiment by Gerdes *et al*. [1] and the manually curated Profiling of E. coli Chromosome (PEC) database (http://www.shigen.nig.ac.jp/ecoli/pec/index.jsp) [2]) to validate orthologous genes (if present) in MR-1. The *Shewanella/Acinetobacter* orthologs were MicrobesOnline tree-orthologs. The *Shewanella/E. coli* orthologs were bidirectional best BLAST hits for which the alignment covered at least 75% of each gene. Also, we took into account whether the putative essential MR-1 genes are contained in the core gene set of the *Shewanella* genus. We defined core genes as those having tree-orthologs [7] in at least 18 of 19 other complete *Shewanella* genomes. Lastly, we considered genomic context in the identification of MR-1 essential genes under the expectation that un-hit genes in an operon with other un-hit or expected essential genes (from *E. coli*) are also likely to be essential. Specifically, we first defined a candidate list of MR-1 essential genes as those that are essential in *E. coli* (Gerdes) or *Acinetobacter* or are unlikely to be un-hit by chance (Poisson $P < 0.05$). Given these candidates, we classified genes as new essential genes if they were candidates, lacked a good insertion, were not classified as essential in *E. coli* (Gerdes or PEC), and were either (1) essential in Acinetobacter and not expected to be auxotrophs; (2) adjacent to and in an operon with another candidate; or (3) core *Shewanella* genes and highly unlikely to be un-hit by chance (Poisson $P < 0.001$). Expected-essential genes were defined as orthologs of *E. coli* essential genes (from Gerdes or PEC) that lacked a good hit.

References

1. Gerdes SY, Scholle MD, Campbell JW, Balazsi G, Ravasz E, et al. (2003) Experimental determination and system level analysis of essential genes in Escherichia coli MG1655. J Bacteriol 185: 5673-5684.
2. Hashimoto M, Ichimura T, Mizoguchi H, Tanaka K, Fujimitsu K, et al. (2005) Cell size and nucleoid organization of engineered Escherichia coli cells with a reduced genome. Mol Microbiol 55: 137-149.
3. de Berardinis V, Vallenet D, Castelli V, Besnard M, Pinet A, et al. (2008) A complete collection of single-gene deletion mutants of Acinetobacter baylyi ADP1. Mol Syst Biol 4: 174.
4. Garrett TA, Kadrmas JL, Raetz CR (1997) Identification of the gene encoding the Escherichia coli lipid A 4'-kinase. Facile phosphorylation of endotoxin analogs with recombinant LpxK. J Biol Chem 272: 21855-21864.
5. Zhang R, Lin Y (2009) DEG 5.0, a database of essential genes in both prokaryotes and eukaryotes. Nucleic Acids Res 37: D455-458.
6. Novichkov PS, Laikova ON, Novichkova ES, Gelfand MS, Arkin AP, et al. (2010) RegPrecise: a database of curated genomic inferences of transcriptional regulatory interactions in prokaryotes. Nucleic Acids Res 38: D111-118.
7. Dehal PS, Joachimiak MP, Price MN, Bates JT, Baumohl JK, et al. (2010) MicrobesOnline: an integrated portal for comparative and functional genomics. Nucleic Acids Res 38: D396-400.
