# Supplementary material for: Evidence-Based Annotation of Gene Function in Shewanella oneidensis MR-1 Using Genome-Wide Fitness Profiling across 121 Conditions
Source: PLoS Genet. 2011 Nov 17;7(11):e1002385. doi: 10.1371/journal.pgen.1002385 (PMC3219624; doi:10.1371/journal.pgen.1002385)
Supplement: Text S2 — Rationale for new gene annotations. (PDF) [file pgen.1002385.s019.pdf]

Text S2: Rationale for new gene annotations

Annotations are new unless indicated otherwise as expanded or confirmed.

SO_0002 (VIMSS 199199) Glutathione uptake transporter
Original annotation: proton/peptide symporter family protein
Comment: This gene is specifically sick with glutathione as the sole source of sulfur. Glutathione is a tripeptide (albeit with an unusual linkage), so this gene may take up other peptides as well.

SO_0444 (VIMSS 199636) Copper/zinc efflux protein
Original annotation: hypothetical protein
Comment: SO_0444 belongs to DUF318 and is a putative membrane protein. The knockout is very sensitive to $CuCl_2$ or $ZnSO_4$ stress. Consistent with this, RegPrecise predicts that SO_0444 is in an operon with and regulated by the upstream gene ZntR, which is expected to regulate zinc and also cadmium efflux (we do not have fitness data for cadmium stress) [1].

SO_0455 and SO_0456 (VIMSS 199647:199648) Alpha-ketoglutarate transporter
Original annotation: hypothetical transporter (SO_0455) and immunogenic-related protein (SO_0456)
Comment: SO_0455 and SO_0456 encode a putative TRAP transporter which we propose is specific for alpha-ketoglutarate, as both genes seem to be required for growth only with alpha-ketoglutarate as the carbon source. Characterized TRAP transporters contain two membrane subunits and a periplasmic component; SO_0455 is a fusion of the two membrane components and SO_0456 is the periplasmic component.

SO_0625 (VIMSS 199813) Cytochrome c oxidase regulatory protein
Original annotation: conserved domain protein
Comment: SO_0625 has high cofitness with the cytochrome c oxidase (cco) operon (SO_2364:SO_2357, most $r > 0.9$). It is likely periplasmic and contains SEL1 repeats, which suggests a regulatory role.

SO_0888 (VIMSS 200074) N-carbamoyl-putrescine amidase
Original annotation: amidase family protein
Comment: The first two steps in the arginine decarboxylase pathway from arginine to putrescine (SO_1870, SO_0887) are sick in media with gelatin as the carbon source. The third step, an amidase reaction, is not annotated. As this gene is adjacent to SO_0887, belongs to an amidase family, and is also sick in the same gelatin media, it is probably the missing N-carbamoyl-putrescine amidase.

SO_1033:SO_1034 (VIMSS 200216:200217) Vitamin B12 transporter components (confirmed)
Original annotation: components of iron compound ABC transporter
Comment: SO_1033 and SO_1034 were originally annotated as components of an ABC transporter for iron compounds, but were proposed by Rodionov et al. [2] to be components of the vitamin B12 transporter. Indeed, they have virtually identical fitness patterns as SO_3709, which is proposed to encode the other (periplasmic) ABC component and is orthologous to E. coli *btuF*, and the proposed *btuB* (SO_0815). Furthermore, they have very similar fitness patterns as other vitamin B12-related genes such as SO_1039 (*cobO*) and SO_1030 (*metH*, a vitamin B12-dependent enzyme).

SO_1115 (VIMSS 200295) Glycine-aspartate dipeptidase (expanded)
Original annotation: aminoacyl-histidine dipeptidase (*pepD*)
Comment: This gene is orthologous to *pepD* from *E. coli*, which is reported to hydrolyse some dipeptides with an unblocked amino terminus [3]. In our data, the gene is specifically required for growth on glycine-aspartate as a carbon source, thus expanding the known range of this enzyme. It may cleave the substrates reported for the *E. coli* enzyme as well; we do not have relevant fitness data.

SO_1267 (VIMSS 200445) gamma-glutamyl-aminobutyrate hydrolase (confirmed)
Original annotation: hypothetical glutamine amidotransferase
Comment: This annotation was corrected in the metabolic model of Pinchuk et al. [4]. Mutants in SO_1267 are specifically sick on putrescine as a nitrogen source, which is consistent with its role in the gamma-gutamyl-putrescine pathway.

SO_1268 (VIMSS 200446) gamma-glutamyl-putrescine synthetase (confirmed)
Original annotation: glutamine synthetase
Comment: This annotation was corrected in the metabolic model of Pinchuk et al. [4]. KEGG annotated it as a glutamate-ammonia ligase. Mutants in SO_1268 are specifically sick on putrescine as a nitrogen source, consistent with its role in the gamma-gutamyl-putrescine pathway.

SO_1270:SO_1273 (VIMSS 200448:200451) Broad range amino acid transporter (expanded)
Original annotation: polyamine ABC transporter
Comment: The ABC transporter operon SO_1270:SO_1273 is annotated as transporting polyamine or putrescine, and it does seem to be required for growth on putrescine. However, insertions in this operon are also very sick on leucine (as sole source of either carbon or nitrogen), alanine, methionine, and possibly D-lactate. It

probably takes up a range of compounds.

SO_1427:SO_1432 (VIMSS 200602:200607) DMSO or manganese oxide reductase (expanded)
Original annotation: DMSO reductase
Comment: DMSO reductase (*dmsEFABGH*, SO_1427:SO_1432) also seems to be the major manganese oxide reductase. These genes are required for growth with DMSO or manganese oxide as the electron acceptor but not in other conditions. Consistent with its role in reducing largely insoluble manganese oxide, DMSO reductase localizes outside of the outer membrane [5].

SO_1521 (VIMSS 200692) D-lactate:flavin oxidoreductase
Original annotation: iron-sulfur cluster-binding protein
Comment: SO_1521 was recently identified as the major D-lactate dehydrogenase in MR-1 by Pinchuk et al. [6]. They suggested that its FeS cluster binding domains might transfer electrons to cytochromes; more recently, a metabolic model [4] by some of the same authors shows it passing electrons to ubiquinone or menaquinone. Our pool data confirms that SO_1521 is required for growth on D-lactate (and also on propionate). Furthermore, we found that NADH-flavin reductase (*fre*, SO_0504) is required for growth on D-lactate but not in most other growth conditions. So, we suggest that SO_1521 passes electrons to flavins, similar to its distant homologs in yeast.

SO_1670 (VIMSS 200835) Fumarylacetoacetate hydrolase (confirmed)
Original annotation: fumarylacetoacetate hydrolase family protein
Comment: SO_1670 is annotated in the metabolic model of Pinchuk et al. [4] but not in KEGG, and the original genome annotation is ambiguous. SO_1670 mutants are very sick on phenylalanine as a nitrogen source and on gelatin. This confirms the annotated function, which is involved in the catabolism of phenylalanine and tyrosine.

SO_1677 (VIMSS 200842) Acetyl-CoA/2-methyl-acetyl-CoA acetyltranferase (expanded)
Original annotation: acetyl-CoA acetyltransferase (*atoB*)
Comment: This gene is required to use isoleucine as a carbon or nitrogen source, which implies that it acts on 2-methylacetyl CoA as well. Without this reaction, 2-methylacetoacetyl-CoA would be a metabolic dead end.

SO_1679 (VIMSS 200844) methylbutyrl-CoA oxidoreductase (confirmed)
Original annotation: acyl-CoA dehydrogenase family protein
Comment: This gene is required for isoleucine utilization. It was annotated by

MetaCyc but not by KEGG or in the metabolic model of Pinchuk et al. [4]. It is also sick when leucine is the carbon source, so it may have another activity.

SO_1683 (VIMSS 200848) Putative 2-methyl-3-hydroxybutyryl-CoA dehydrogenase
Original annotation: 3-oxoacyl-(acyl-carrier-protein) reductase, putative
Comment: We do not have fitness data on this gene, but this activity is required to explain the isoleucine degradation data, and this gene is in a cluster with other genes for catabolism of branched-chain amino acids.

SO_1854 (VIMSS 201016) Outer membrane protein required for motility and nitrate resistance
Original annotation: hypothetical protein
Comment: SO_1854 belongs to DUF3466 and localizes to the outer membrane [7]. Knockouts in this gene are non-motile and sensitive to ammonium nitrate. As with many motility-related genes, knockouts in SO_1854 appear to have an advantage in most growth conditions.

SO_1913 (VIMSS 201074) Chaperone for general secretory pathway
Original annotation: conserved hypothetical protein
Comment: SO_1913 has a complex fitness pattern -- it is required for motility and also for growth on a few carbon and nitrogen sources and in some stress experiments. SO_1913 has a very similar fitness pattern as the *gsp* operon (SO_0165:SO_0176, most pairwise $r > 0.8$). These genes also have similar fitness patterns as the leader processing enzyme *pilD* (SO_0414); however, SO_1913 and the *gsp* system are required in a few growth conditions where *pilD* is not required (e.g., butyrate or DNA as the carbon source). Thus it appears that SO_1913 is required for most activities of the *gsp* system; furthermore, it is required independently of *pilD*. SO_1913 is similar to an uncharacterized E. coli protein (*ybaY*, predicted outer membrane lipoprotein), and it has weak similarity to a chaperone involved in type III secretion (PF09619.3, 15.5 bits, E < 1e-5, HMMer 3 without heuristic search and no correction for multiple testing). We propose that SO_1913 is a chaperone for the general secretory pathway. We also note that some homologs of SO_1913 are fused to hslJ/META domains -- the molecular function of these domains is unknown but they are implicated in heat shock in diverse organisms.

SO_1916 (VIMSS 201077) DMSO-specific transcriptional activator of SO_1917
Original annotation: transcriptional regulator, LysR family
Comment: SO_1916 and the adjacent gene SO_1917 have similar fitness patterns ($r = 0.56$). SO_1916 has strong phenotypes only when SO_1917 did as well, suggesting that SO_1917 might be the primary target. Both genes are sick when DMSO is the electron acceptor but not on lactate/O_2. Expression data confirmed activation of

SO_1917 by SO_1916 under DMSO reducing but not O_2 reducing conditions.

SO_1971 (VIMSS 201132) Butyryl-CoA synthase
Original annotation: AMP-binding family protein
Comment: This gene is annotated by KEGG as an acyl-CoA synthase, but was not included in the metabolic model of Pinchuk et al. [4]. It is specifically required for growth on butyrate as a carbon source. Oddly, another fatty-CoA synthase *fadD-2* (SO_3664) is also sick on butyrate, but mutants in SO_1971 are less fit (-2.4 vs. -1.5). The genes may be partially redundant or *fadD-2* might have some other role.

SO_2357 (VIMSS 201501) Cytochrome c oxidase maturation protein
Original annotation: conserved hypothetical protein
Comment: SO_2357 belongs to COG2836 and to PFam PF02683 (disulfide bond interchange family dbsD) and matches the fitness pattern of the other (upstream) genes in the cco operon.

SO_2395 (VIMSS 201539) Butyryl-CoA dehydrogenase
Original annotation: acyl-CoA dehydrogenase family protein
Comment: This gene is required for growth on butyrate as a carbon source but is beneficial to lose on most other conditions. RegPrecise predicts SO_2395 repression by PsrA [1], which regulates fatty acid catabolism and which is beneficial to lose on butyrate.

SO_2593 (VIMSS 201733) NAD amino acid dehydrogenase (expanded)
Original annotation: conserved hypothetical protein
Comment: In the metabolic model of Pinchuk et al. [4], SO_2593 is proposed to be NAD-glutamate dehydrogenase (GudA) and to be an optimal pathway for incorporation of ammonia. However, our data suggests that glutamate synthase (*gtlBD*, SO_1324:SO_1325) is required for growth unless glutamine, glutamate, or asparate is provided. In contrast, SO_2593 is not required for aerobic growth on minimal lactate media. Instead, SO_2593 is sick on LB and when any one of a variety of amino acids is the sole sources of carbon or nitrogen. So, we suggest that SO_2593 reacts with a wide range of amino acids and that its main function is catabolic. A potential issue in interpreting our pool data for SO_2593 is that it is upstream of and potentially polar on *pyrD*, a pyrimidine auxotroph. However, SO_2593 and *pyrD* have only modest correlations in their patterns of gene expression (r = 0.35 on MicrobesOnline) or fitness (r = 0.20).

SO_2638 (VIMSS 201776) branched-chain amino acid dehydrogenase (confirmed)
Original annotation: leucine dehydrogenase (*ldh*)

Comment: In the metabolic model of Pinchuk et al. [4], SO_2638 is proposed to act on isoleucine and valine as well as leucine. We do not have data for valine, but SO_2638 is required for utilizing leucine or isoleucine as a carbon source. Surprisingly, it is not involved in using leucine as a nitrogen source and is only a bit sick on isoleucine as a nitrogen source.

SO_2648 (VIMSS 201786) Reponse regulator and transcriptional activator of Acetyl-CoA synthase
Original annotation: DNA-binding response regulator, LuxR family
Comment: Mutants in SO_2648 have highly correlated fitness with mutants in SO_2742 (r = 0.87) which is adjacent to Acetyl-CoA synthase (SO_2743). Expression data confirmed that this gene is required for expression of Acetyl-CoA synthase.

SO_2742 (VIMSS 201880) Histidine kinase targeting SO_2648
Original annotation: sensor histidine kinase/response regulator
Comment: Contains a potential coenzyme-A binding or symporter domain at its N terminus. We propose that SO_2648 targets SO_2742 which then regulates Acetyl-CoA synthase (SO_2743). Expression data from a mutant in this gene after transfer to acetate was similar to that of a mutant in SO_2648 (r = 0.79) and higher than the similarity to a control (a mutant in transcriptional regulator SO_1669, which also does not grow on acetate, r=0.50-0.66).

SO_2846 (VIMSS 201972) Glycine transport protein
Original annotation: conserved hypothetical protein
Comment: SO_2846 is similar to sulfite and sulfoacetate exporters (PFam PF01925) and is orthologous to E. coli *yfcA*, which encodes an inner membrane protein of unknown function. Mutants in SO_2846 are very sick with glycine as the nitrogen source, so we suggest that it is a glycine transporter.

SO_2879 (VIMSS 202005) N-acetylglucosamine and uracil permease
Original annotation: uracil permease (uraA)
Comment: Knockouts in this gene are specifically sick with N-acetylglucosamine (NAG) as the sole source of nitrogen. Although the MR-1 genome encodes another NAG transporter NagP (SO_3503), *nagP* mutants are not sick with NAG as the nitrogen source, which suggests that there is another transporter (*nagP* mutants are indeed sick with NAG as the carbon source under both aerobic and anaerobic conditions). One possibility is that *nagP* is poorly expressed in the presence of lactate. Gene order suggests that SO_2879 also acts as a uracil permease, but we have no relevant fitness data.

SO_3102:SO_3103 (VIMSS 202218:202219) Thiophosphate efflux pump

components
Original annotation: AcrAB family proteins
Comment: SO_3102 and SO_3103 are putative AcrAB-like efflux pump components that are specifically sick with thiophosphate as the sole source of sulfur. We propose that these efflux pump components keep thiophosphate from building up in the cell, where it may attack cysteine residues. These components are expected to work together with TolC (SO_3904), which is required for growth on thiophosphate but not on sulfate or sulfite. (*tolC* mutants are sick in many other conditions as well.) Thiophosphate may be cleaved to sulfide and phosphate by non-specific alkaline phosphatases [8], which we are probably present in the periplasm or secreted, as the MR-1 genome encodes at least five alkaline phosphatases.

SO_3175 (VIMSS 202285) Cell wall component synthesis enzyme
Original annotation: asparagine synthetase, glutamine-hydrolyzing (*asnB-2*)
Comment: This gene is in a cell wall synthesis operon and is required for motility but not for growth on minimal media. Its paralog *asnB-1* (SO_2767) is an auxotroph, as expected if SO_3175 has another function. We propose that SO_3175 transfers an amine group onto a precursor of the cell wall.

SO_3259:SO_3260 (VIMSS 202367:202368) Flagellar modification gene cluster
Original annotation: Conserved hypothetical genes
Comment: The gene cluster SO_3259:SO_3260 is adjacent to a flagellar gene cluster and is required for motility. These genes were originally annotated as hypothetical proteins. SO_3259 is similar to flagellar modification genes *pseD* and *pseE* from *Campylobacter jejuni*, which are involved in decorating the flagellum with sugars and are required for full motility [9]. Thus, we propose that this cluster encodes the modification of the flagellum by some sugar.

SO_3262 (VIMSS 202370) Polysaccharide synthesis gene
Original annotation: acetolactate synthase isozyme I, large subunit (*ilvB*)
Comment: This gene is in a polsaccharide biosynthesis operon (SO_3261:SO_3265) and is required for motility but not for growth on minimal media. Unlike the adjacent genes SO_3259 and SO_3260, SO_3262 (like other genes of the operon) has phenotypes in conditions other than motility. We are not sure whether the motility phenotype of SO_3262 is related to the modification of the flagellum (as for SO_3259:SO_3260) or a cell wall component (which may reduce motility by altering cell morphology). The MR-1 genome includes another acetolactate synthase (*ilvMG*), which is sick unless amino acids are provided.

SO_3496 (VIMSS 202599) succinate-semialdehyde:NAD dehydrogenase (confirmed)
Original annotation: aldehyde dehydrogenase

Comment: The metabolic model of Pinchuk et al. [4] gives the specific annotation, while KEGG and the original genome annotation have the generic annotation. This gene is required specifically for the utilization of putrescine as a nitrogen source, consistent with its role in the gamma-glutamyl-putrescine pathway.

SO_3635 (VIMSS 202731) Cell wall phosphotransferase required for survival in late stationary phase
Original annotation: hypothetical phosphotransferase
Comment: Mutants in this gene have greatly reduced survival 60 hours after the onset of stationary phase. The gene is in a conserved operon with putative LPS-related gene (SO_3634) and contains a phosphotransferase domain (PF01636).

SO_3749 (VIMSS 202842) N-acetyl-L-ornithine deacetylase
Original annotation: hypothetical protein
Comment: This gene is an arginine auxotroph, which suggests that it performs the unannotated step in arginine synthesis to convert N-acetyl-L-ornithine to ornithine. It could cleave its substrate to ornithine and acetate or it could transfer the acetyl group elsewhere. The protein has weak homolgy to asparatoacylases, which suggests that it is a deacetylase.

SO_4008 (VIMSS 203092) Recombination regulatory protein
Original annotation: hypothetical protein
Comment: Mutants in SO_4008 have highly correlated fitness with mutants in *rdgC* (SO_1556; r = 0.92). Both genes lack strong phenotypes in most conditions but are sensitive to high temperature, are not motile, and are sick with threonine as the sole source of nitrogen. In *E. coli*, RdgC binds DNA and regulates recombination by competing with RecA for binding. SO_4008 belongs to the uncharacterized family DUF3584 and is homologous to Smc (structural maintenance of chromosomes) proteins (COG0419) that regulate DNA segregation or recombination and contain ATPase domains. SO_4008 also contains a divergent P-loop NTPase domain (SSF52540, model 0036790, 18.0 bits, E < 1e-6 , HMMer 3 without heuristic search and no correction for multiple testing). Overall, the sequence analysis confirms that SO_4008 and RdgC have related functions, so we propose that SO_4008 also binds DNA and regulates recombination.

SO_4198 (VIMSS 203280) Formiminoglutaminase (confirmed)
Original annotation: arginase family protein
Comment: SO_4198 was annotated as formiminoglutaminase in the metabolic model of Pinchuk et al. [4]. Consistent with the revised annotation, SO_4198 is required for utilization of histidine as a nitrogen source.

SO_4339 (VIMSS 203417) Hypotaurine transporter
Original annotation: sodium-dependent transporter, putative
Comment: A knockout in this gene is specifically sick with hypotaurine as the sole source of sulfur.

SO_4485 (VIMSS 203560) Diheme cytochrome c for energy production
Original annotation: diheme cytochrome c
Comment: Genes of this family (PF09626) are proposed to transfer electrons to Shp [10]. SO_4485 is adjacent to *shp* (SO_4484) but *shp* mutants lack strong phenotypes in our data. In contrast, a SO_4485 mutant is sick on most aerobic growth conditions and some anaerobic conditions. Apparently SO_4485 has some other role in energy generation.

SO_4565 (VIMSS 203636) L-leucine transporter
Original annotation: inner membrane protein YjeH
Comment: Knockouts in this gene are very sick with L-leucine as the sole source of nitrogen, and are also sick with L-leucine as the sole source of carbon. SO_4565 is orthologous to an uncharacterized inner membrane protein from *E. coli* (YjeH) and belongs to a family of amino acid transporters (COG-PotE).

References

1. Novichkov PS, Laikova ON, Novichkova ES, Gelfand MS, Arkin AP, et al. (2010) RegPrecise: a database of curated genomic inferences of transcriptional regulatory interactions in prokaryotes. Nucleic Acids Res 38: D111-118.
2. Rodionov DA, Vitreschak AG, Mironov AA, Gelfand MS (2003) Comparative genomics of the vitamin B12 metabolism and regulation in prokaryotes. J Biol Chem 278: 41148-41159.
3. Schroeder U, Henrich B, Fink J, Plapp R (1994) Peptidase D of Escherichia coli K-12, a metallopeptidase of low substrate specificity. FEMS Microbiol Lett 123: 153-159.
4. Pinchuk GE, Hill EA, Geydebrekht OV, De Ingeniis J, Zhang X, et al. (2010) Constraint-based model of Shewanella oneidensis MR-1 metabolism: a tool for data analysis and hypothesis generation. PLoS Comput Biol 6: e1000822.
5. Gralnick JA, Vali H, Lies DP, Newman DK (2006) Extracellular respiration of dimethyl sulfoxide by Shewanella oneidensis strain MR-1. Proc Natl Acad Sci U S A 103: 4669-4674.
6. Pinchuk GE, Rodionov DA, Yang C, Li X, Osterman AL, et al. (2009) Genomic reconstruction of Shewanella oneidensis MR-1 metabolism reveals a previously uncharacterized machinery for lactate utilization. Proc Natl Acad Sci U S A 106: 2874-2879.

7. Elias DA, Monroe ME, Marshall MJ, Romine MF, Belieav AS, et al. (2005) Global detection and characterization of hypothetical proteins in Shewanella oneidensis MR-1 using LC-MS based proteomics. Proteomics 5: 3120-3130.
8. Chlebowski JF, Coleman JE (1974) Mechanisms of hydrolysis of O-phosphorothioates and inorganic thiophosphate by Escherichia coli alkaline phosphatase. J Biol Chem 249: 7192-7202.
9. Ewing CP, Andreishcheva E, Guerry P (2009) Functional characterization of flagellin glycosylation in Campylobacter jejuni 81-176. J Bacteriol 191: 7086-7093.
10. Gibson HR, Mowat CG, Miles CS, Li BR, Leys D, et al. (2006) Structural and functional studies on DHC, the diheme cytochrome c from Rhodobacter sphaeroides, and its interaction with SHP, the sphaeroides heme protein. Biochemistry 45: 6363-6371.
